# Neuroprotective effect of the RNS60 in a mouse model of transient focal cerebral ischemia

**DOI:** 10.1371/journal.pone.0295504

**Published:** 2024-01-02

**Authors:** Gloria Patricia Baena-Caldas, Jie Li, Lina Pedraza, Supurna Ghosh, Andreas Kalmes, Frank C. Barone, Herman Moreno, A. Iván Hernández

**Affiliations:** 1 Departments of Neurology and Physiology & Pharmacology, SUNY Downstate Health Sciences University, Brooklyn, NY, United States of America; 2 Department of Pathology, SUNY Downstate Health Sciences University, Brooklyn, NY, United States of America; 3 Health Sciences Division, Department of Morphology, School of Biomedical Sciences, Universidad del Valle, Cali, Colombia; 4 Revalesio Corporation, Tacoma, WA, United States of America; 5 The Robert F. Furchgott Center for Neural and Behavioral Science, Downstate Medical Center, State University of New York, Brooklyn, NY, United States of America; Massachusetts General Hospital/Harvard Medical School, UNITED STATES

## Abstract

**Background:**

Stroke is a major cause of death, disability, and public health problems. Its intervention is limited to early treatment with thrombolytics and/or endovascular clot removal with mechanical thrombectomy without any available subacute or chronic neuroprotective treatments. RNS60 has reduced neuroinflammation and increased neuronal survival in several animal models of neurodegeneration and trauma. The aim here was to evaluate whether RNS60 protects the brain and cognitive function in a mouse stroke model.

**Methods:**

Male C57BL/6J mice were subjected to sham or ischemic stroke surgery using 60-minute transient middle cerebral artery occlusion (tMCAo). In each group, mice received blinded daily administrations of RNS60 or control fluids (PNS60 or normal saline [NS]), beginning 2 hours after surgery over 13 days. Multiple neurobehavioral tests were conducted (Neurological Severity Score [mNSS], Novel Object Recognition [NOR], Active Place Avoidance [APA], and the Conflict Variant of APA [APAc]). On day 14, cortical microvascular perfusion (MVP) was measured, then brains were removed and infarct volume, immunofluorescence of amyloid beta (Aβ), neuronal density, microglial activation, and white matter damage/myelination were measured. SPSS was used for analysis (e.g., ANOVA for parametric data; Kruskal Wallis for non-parametric data; with post-hoc analysis).

**Results:**

Thirteen days of treatment with RNS60 reduced brain infarction, amyloid pathology, neuronal death, microglial activation, white matter damage, and increased MVP. RNS60 reduced brain pathology and resulted in behavioral improvements in stroke compared to sham surgery mice (increased memory-learning in NOR and APA, improved cognitive flexibility in APAc).

**Conclusion:**

RNS60-treated mice exhibit significant protection of brain tissue and improved neurobehavioral functioning after tMCAo-stroke. Additional work is required to determine mechanisms, time-window of dosing, and multiple dosing volumes durations to support clinical stroke research.

## Introduction

Due to its global prevalence and the costs of treatment and disability, stroke is one of the most serious public health problems in modern times [1–3]. There are two main causes of stroke: ischemic and hemorrhagic. Ischemic strokes account for 85–87% of all cases [4, 5]. Currently, acute ischemic stroke therapies aim to dissolve or remove the clot using tissue plasminogen activator (tPA) and/or endovascular interventional procedures such as mechanical thrombectomy [6–9]. These therapies are limited by a short time window for intervention, strict patient selection criteria, high risk complications, and high costs [9–11]. To date, there are no effective treatments for the subacute and chronic phases that follow acute cerebral ischemia as a direct result of ischemic stroke, or as a response to reperfusion after tPA therapy and/or endovascular intervention [12, 13]. The treatment limitations in all post-stroke phases necessitate a search for new therapies, including the possible use of cerebroprotective agents that reduce brain loss [14–17]. Transient middle cerebral artery occlusion (tMCAo) is used as a model of ischemic stroke [18, 19]. In mice, 60 minutes (min) of tMCAo leads to acute severe hypoxia followed by a complex cascade of progressive pathophysiological events that appear over the course of hours (h) to months after reperfusion [12, 20]. These events include blood-brain barrier impairment, white matter damage, necrosis, inflammation, cellular damage (energy failure, increase in reactive oxygen species (ROS), oxidative stress, free radical production), intracellular and extracellular aggregation of neurodegenerative proteins that lead to loss of neuronal function and apoptosis, abnormal neurogenesis, motor alterations, and cognitive deterioration [21, 22].

RNS60 is a physically modified saline that is generated by subjecting 0.9% normal saline to Taylor-Couette-Poiseuille (TCP) flow under elevated oxygen pressure. Chemically, RNS60 contains water, sodium chloride, and 50–60 parts/million oxygen [23, 24]. RNS60 has been studied as a potential treatment in various neurodegenerative disease models including multiple sclerosis, amyotrophic lateral sclerosis, traumatic brain injury, as well as Alzheimer’s and Parkinson’s diseases. In these studies, it has shown anti-inflammatory and anti-apoptotic properties resulting in a reduction of tissue damage [25–29]. Here we have extended the investigation of RNS60 as a treatment of stroke in C57BL/6J mice to assess whether RNS60 has a positive effect on prevention and/or recovery from tissue and cognitive damage after ischemic stroke.

## Materials and methods

All procedures were approved by the SUNY Downstate Institutional Animal Care and Use Committee (IACUC) under protocol number 15–10475. All efforts were made to minimize the number of animals used and their suffering.

### Animals

Seventy-six young-adult (3–4 months old) C57BL/6J male mice (Jackson Lab; Bar Harbor, ME, USA) were randomized into the study using the standard = RAND() function in Microsoft Excel. Postoperative exclusion criteria were inability to eat, drink, or ambulate normally, loss in body weight ≥ 15%, bleeding, and death. One animal experienced post-surgical complication and postoperative death (autopsy showed hemorrhage during thread insertion). A total of 75 mice were therefore included in the study and were kept in single cages inside the SUNY Downstate animal facility at 22 ± 2°C, with a 12–12 light cycle and food and water ad libitum, throughout the study. All the behavioral experiments were performed during daylight from ZT 0—ZT 4 (7 AM-11 AM). All the sacrifices/tissue and fluid collections were made between ZT 0 –ZT 6 (7 AM- 1 PM).

Animals were observed daily by investigators and the veterinary staff of the Division of Comparative Medicine (DCM) of SUNY Downstate Health Sciences University.

### Animal model of stroke (surgical procedures)

Middle cerebral artery occlusion (tMCAo, n = 42) or sham surgeries (n = 33) were conducted as previously described [30]. To reduce variability during surgery, we used animals with a weight between 18–22 g and a special suture that was developed for mice described by Lemmerman et al [31]. In our experience, the monofilament suture 6–0 (Doccol Corp, Sharon, MA, USA) has a very good fit in the origin of the medial cerebral artery of animals of that weight. Under isoflurane anesthesia (1–2%), the monofilament suture 6–0 (Doccol Corp, Sharon, MA, USA) was inserted through the proximal left external carotid artery, advanced into the left internal carotid artery, and positioned to occlude the origin of the left middle cerebral artery.

After 60 min of occlusion, the intraluminal filament was withdrawn to allow reperfusion. Body temperature was continuously maintained at 37.0 ± 0.5°C during and after surgery using Adroit Medical System’s Heat Therapy Pump-1500 and Temperature Therapy Pad (Louden, TN, USA) until animals completely recovered from anesthesia. Animals were then placed in housing cages, monitored closely over the next 4 h and then daily for the rest of the study. The sham operated mice were subjected to the same procedures and handling except for the occlusion. Relative to other ischemic stroke models, intraluminal tMCAo offers improved reproducibility in terms of infarct volume and cognitive/functional deficits, in addition to not requiring craniotomy [31].

### Experimental treatment

The animals were treated with once daily intraperitoneal injections of 200 μL of RNS60 or control fluids normal saline (NS) or pressurized oxygenated saline with a similar oxygen content as RNS60 (PNS60) starting 1 h after reperfusion. We selected the RNS60 dose based on previously reported data in other murine neurodegenerative disease models [34]. The investigators remained blinded to the treatment throughout the study.

Six groups of animals (n = 9 per group) were treated for 13 days (S1 Fig) with RNS60, NS or PNS60. We estimated the size sample to be sufficient to achieve statistical significance (p<0.05) with sufficient power (0.08) based on previous theoretical calculations and experience [30, 32, 33]. The random allocation of mice to experimental group were generated using the standard = RND() function in Microsoft Excel. Twelve mice without treatment (6 per procedure) were used as control no treatment groups.

For the assessment of hypoxia in the peri-infarct-penumbra a small cohort of animals (n = 3 per group) were treated with daily intraperitoneal (IP) injections (200 μL) of RNS60 or control fluids (NS or PNS60) after tMCAo surgery with daily injections starting 1 h after the onset of reperfusion, ending on day 3. The animals were euthanized on day 4 (S2 Fig). This small cohort was used to determine if RNS60 treatment reduces hypoxia within the peri-infarct-penumbra compared to control solutions.

We selected our dose of treatment based on previous reported data in mice with multiple doses of RNS60 described by Mondal et al. [34].The investigators remained blinded to the treatment throughout the experiment.

### Stroke validation

We used (a) functional and (b) structural stroke validation (for details see Materials and methods in S2 File).

### Cognitive evaluation

See S1 Fig for corresponding timeline.

#### (a)Novel Object Recognition (NOR) test

Animals without cognitive problems prefer exploring new objects if an old and a new object are presented simultaneously, the animals tend to explore the new one [35]. The NOR test was conducted over 4 days: On days 7 and 8, mice were placed in the arena for 5 min without objects (habituation). On day 9, they were placed in the arena for an initial 5 min without objects followed by 5 min with two identical objects. On day 10, the animals were placed in the arena for 5 min with the familiar object used on day 9 and a new one. Memory was tested by Discrimination Index [36].

#### (b)Active Place Avoidance (APA) training

Mice were placed in a rotating (1 rpm) 40-cm circular arena and trained to avoid a nonrotating 60-degree shock zone (see S3A Fig). Whenever they entered the shock zone for at least 500 milliseconds (ms), they received a constant current foot-shock (60 Hz, 0.2 mA, 500 ms). The shock was repeated every 1500 ms until the mouse left the shock zone. The animals learned to avoid the zone using distal spatial cues set outside the arena. APA was performed on days 11 and 12 (4 10-min trials 30 min apart) to assess regular learning and memory. On day 13, the APA conflict was performed (4 10-min trials 30 min apart) for which the shock zone was placed on the opposite side of the initial location (S3B–S3D Fig). The variables evaluated per trial were maximum time avoidance (MTA), time to the first entry (T1stE) into the shock zone, distance, and speed. The MTA to entry into the shock area measures learning within a single session (the ability to actively avoid the shock zone by paying attention to the visual cues), whereas T1stE into the shock zone measures memory from the prior session(s).

### Cerebral microvascular perfusion

On day 14, immediately before euthanasia, cerebral microvascular perfusion (MVP) was assessed by laser speckle contrast imaging (LSCI) (PeriCam PSI High Resolution System; Perimed; Stockholm, Sweden). LSCI system allowed to get both real-time graphs and video recordings of the cerebral blood perfusion measured as arbitrary perfusion units (PU) using an invisible near infra-red laser (wavelength 785 nm, PeriCam PSI system, Perimed, Järfälla, Sweden), and a visible red laser (650 nm) to indicate the maximum measurement area. Image acquisition rate was 25 images/s, and the distance between the laser head and the mouse skull surface was fixed at 10 cm. The resolution was 0.02 mm, and the resulting images were ~0.7 mm in size. The manufacturer’s software (PIMSoft, Perimed, Järfälla, Sweden) was used to analyze the images. Prior to the measurements, anesthetized (isoflurane 1–2%) mice underwent microscopic surgery where an incision was made in the midline of the scalp to expose the surface and cranial sutures of the mouse’s skull and meticulously dissect the skin with a scalpel to position the laser 1 cm posterior to the bregma anatomical point (S4A Fig). Then, neck dissection was performed to expose the right common carotid artery (rCCA), which was occluded with a clamp for 10 s and then released to allow brain reperfusion. LSCI was used to measure MVP in motor cortex (MC) and parietal cortex (PC) with real time recordings which included: 3 min of cerebral basal blood flow (baseline) (S4B and S4C Fig); 10 s transient right common carotid artery occlusion (rCCAo) (S4B and S4D Fig); and a 3 min recovery (S4B and S4E Fig). Circular regions of interest (ROIs) were located in the primary motor cortex-M1 (Br:1.98, In:5.78) and the lateral parietal cortex (Br:-2.06, In:1.74) on both sides of the brain based on the Paxinos’ Mouse Brain Atlas [37]. The ROIs were named: 1. Left parietal cortex (1LPC), 2. Right parietal cortex (2RPC), 3. Left motor cortex (3LMC) and 4. Right motor cortex (4RMC) (S4F and S4G Fig). The baseline was used to identify the differences of cerebral blood flow (CBF) between both cerebral hemispheres in mice subjected to tMCAo and Sham surgery that were injected or not with PNS60, RNS60 or NS treatments. The difference between baseline and occlusion (Δ Downfall) and its percentage of change (% Δ Downfall) were measured to determine whether contralateral and ipsilateral circulation change with different treatments.

### Perfusion, fixation, and immunofluorescence

Described in Supplemental Methods (S2 File).

### Statistical analysis

An unbiased blinded study design was implemented where the solutions were named A, B, and C, until all the data were analyzed. The data analyzed are available on Supplemental Tables in S1 File. Normal distribution and homogeneity of variance were determined using the Shapiro-Wilk normality and Levene’s tests. Data meeting the assumptions of normality and homogeneity of variance were evaluated using one-way or two-way ANOVA followed by Tukey. Data not meeting the assumptions were analyzed using the Kruskal-Wallis test followed by Bonferroni post hoc test. Statistical analyzes were performed with the SPSS program (SPSS version 25, IBM, Armonk, NY, USA). Statistical significance was accepted at p<0.05.

## Results

### Unilateral tMCAo produced a decline in motor performance 24 h after surgery

We tested motor performance before and after tMCAo stroke. At baseline (day 0), 24 h prior to surgical procedures, modified neurological severity score (mNSS) = 0, beam balance (BB) = 0, and foot fault (FF) = 0, were assessed. 24 h after surgery (day 2), mice subjected to left tMCAo exhibited worsened mNSS, BB and FF compared to baseline (all the parameters for the different solutions, PNS60, RNS60 and NS, have a Friedman Test with X^2^_(1)_ = 8.000, p = 0.005). When comparing the tMCAo mice treated with different solutions, there was a trend towards better performance in mice treated with RNS60 (mNSS = 4.6+/-0.9, BB = 2.1+/-0.4 and FF = 19+/-2.8) compared to mice treated with PNS60 (mNSS = 6.3+/-0.6, BB = 2.6+/-0.5 and FF = 23+/-3.4) or NS (mNSS = 6.9+/-1.3, BB = 3.5+/-0.7 and FF = 24+/-4.3) but the effect was not statistically significant at this early time point (see S5 Fig).

### RNS60 treatment reduced infarct areas in animals subjected to unilateral tMCAo

H&E staining showed the infarct area caused by tMCAo and reperfusion after 14 days. Mice treated with RNS60 had significantly smaller (p<0.001) infarct areas compared to both controls showing that RNS60 reduced tissue damage after ischemic stroke. PNS60-treated mice had the largest infarcts (S6 Fig and S1 Table in S1 File).

### RNS60 treatment reduced hemispheric loss in animals subjected to left tMCAo

**Fig 1:**
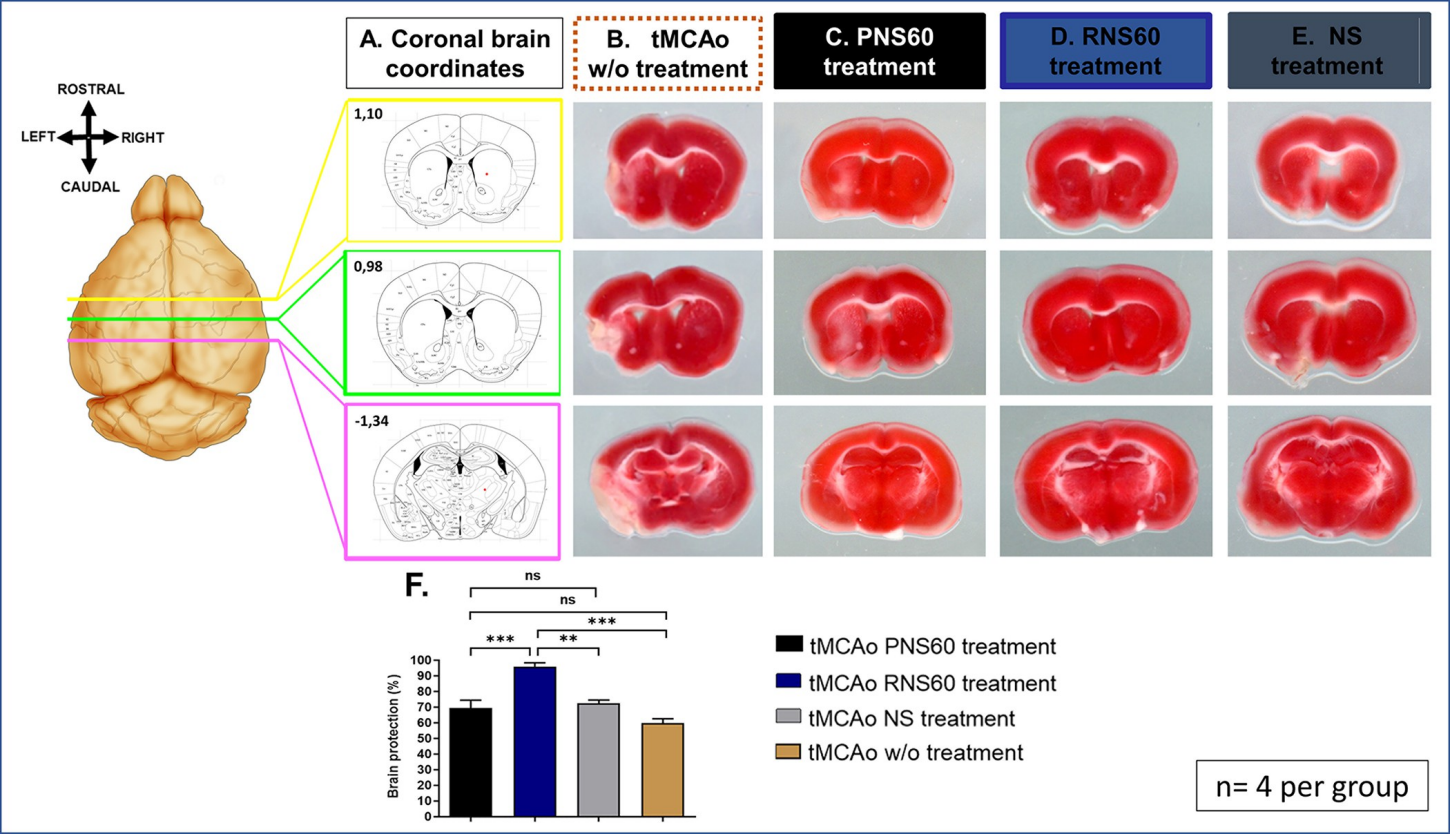
RNS60 protected against hemispheric loss in mice subjected to left tMCAo. A) The cerebral slices of the coordinates 1.10, 0.98 and -1.34 from the Paxinos mouse atlas were used for analysis with ImageJ software. B-E) Representative TTC images of tMCAo brain slices (coordinates shown in Fig A) of untreated (B) mice or mice treated with PNS60 (C), RNS60 (D) or NS (E). F) Quantification of the TTC staining shows increased ipsilateral protection of brain loss in tMCAo mice treated with RNS60 (blue bar) compared to those treated with PNS60 (black bar) or NS (gray bar) as well as mice without any treatment (brown bar). The percentage of protection is shown as mean ± SEM. **p < 0.01, ***p < 0.001 and ns = no statistical significance.

On day 14 after left tMCAo, the brain volume of the ipsilateral hemisphere (compared to the contralateral hemisphere) was calculated using TTC staining (n = 4; 3 brain slices were analyzed for each mouse). There was an approximately 40% hemispheric brain reduction in tMCAo mice without treatment. In contrast, tMCAo mice treated with RNS60 were significantly protected against brain loss compared to PNS60- and NS-treated mice (see Fig 1 and S2 Table in S1 File).

### Cognitive evaluation

To assess whether RNS60 influences cognitive performance after left tMCAo, we evaluated animal performance on NOR and APA.

### Unilateral tMCAo mice treated with RNS60 preferred a novel object in the NOR test

**Fig 2:**
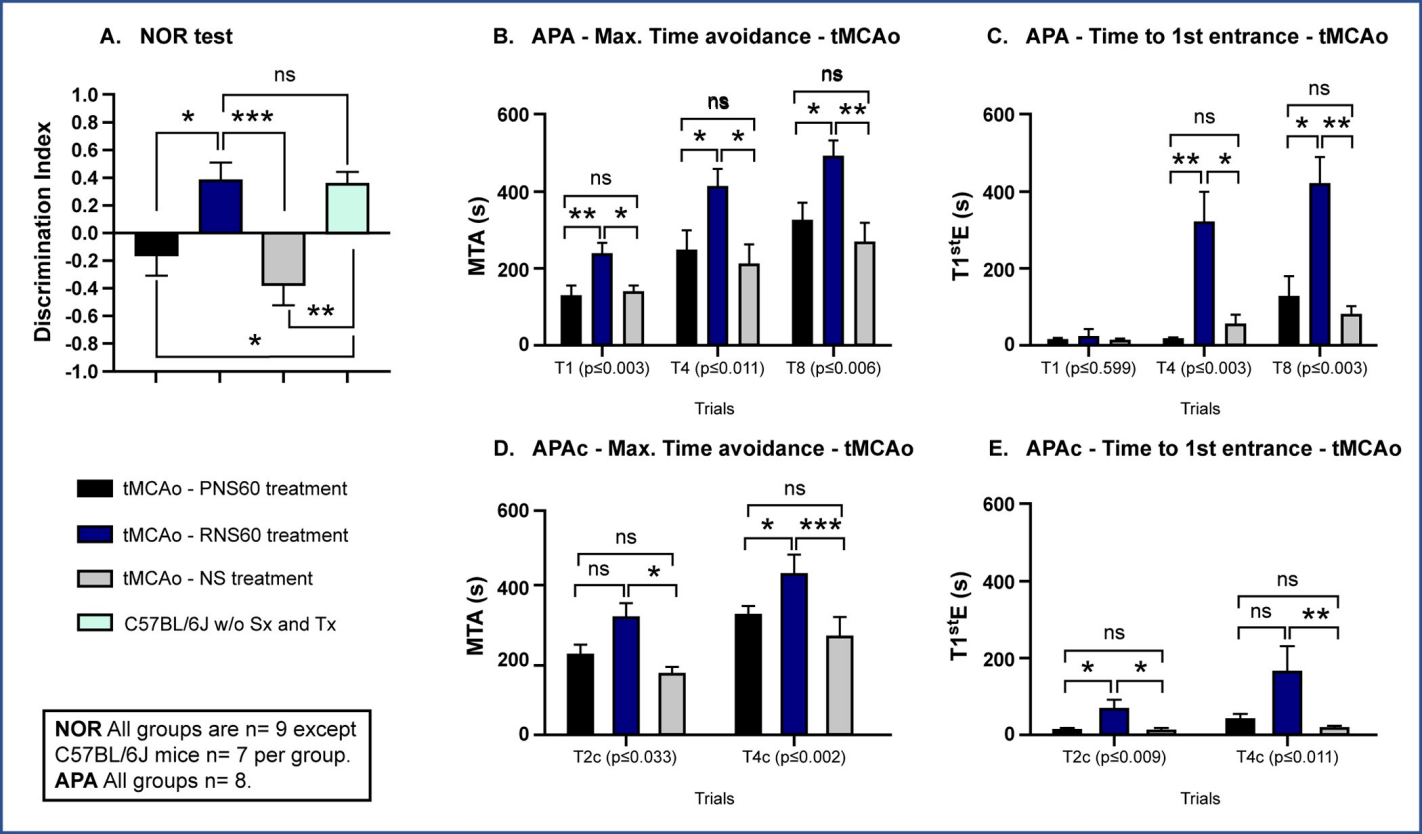
RNS60 treatment improved performance in cognitive evaluation tests. A) NOR task showed that mice subjected to left tMCAo and treated with RNS60 (blue bar) performed similarly to healthy C57BL/6J mice without surgery and treatment (w/o Sx and Tx, green bar). In contrast, tMCAo mice treated with PNS60 (black bar) showed no preference for the new object (discrimination index near 0) suggesting that memory is impaired, and mice with left tMCAo and treated with NS (grey bar) responded with an aversion to the new object (negative discrimination index). B-E) Active place avoidance memory task shows that animals treated with RNS60 have better memory and cognitive flexibility as assessed by APA (B, C) and APA conflict (D, E) tests using two parameters of learning and memory: Maximum Time Avoidance (MTA) (B, D), and time to the 1^st^ entrance (T1^st^E) (C, E). *p<0.05, **p<0.01, ***p<0.001, ns = non-significant.

NOR training was performed during the subacute phase of post ischemic stroke (days 7–10) in tMCAo mice treated with the different solutions compared to healthy mice without surgery or treatment. Mice treated with RNS60 spent more time exploring the new object compared to the known one (Fig 2A). In fact, the NOR test showed that tMCAo mice treated with RNS60 performed similarly to healthy control mice without surgery or treatment, suggesting that treatment with RNS60 produced a good recovery after tMCAo (see Fig 2A and S3 Table in S1 File). When speed and distance were tested, no significant differences were observed among groups suggesting that motor skills were not affecting the performance (see S10A and S10B Fig, and S13 Table in S1 File).

### tMCAo mice treated with RNS60 exhibited improved APA performance

The APA test was used to compare cognitive performance. We found that tMCAo mice treated with RNS60 performed better in parameters of maximum time avoidance (MTA) and time to the first entrance (T1stE) on days 11 (trials 1–4) and 12 (trials 5–8) suggesting a recovery of learning and memory (Fig 2B and 2C and S4 and S5 Tables in S1 File). On day 13, when cognitive flexibility was tested (APA conflict), the improvement was milder and only clearly observed in trial 4 of MTA (trials 2c – 4c; Fig 2D and 2E and S4 and S5 Tables in S1 File). When speed and distance were tested, no significant differences were observed among groups suggesting that motor skills were not affecting the performance (see S10C–S10F Fig and S14 and S15 Tables in S1 File).

### RNS60 treatment increased cortical cerebral microvascular perfusion

**Fig 3:**
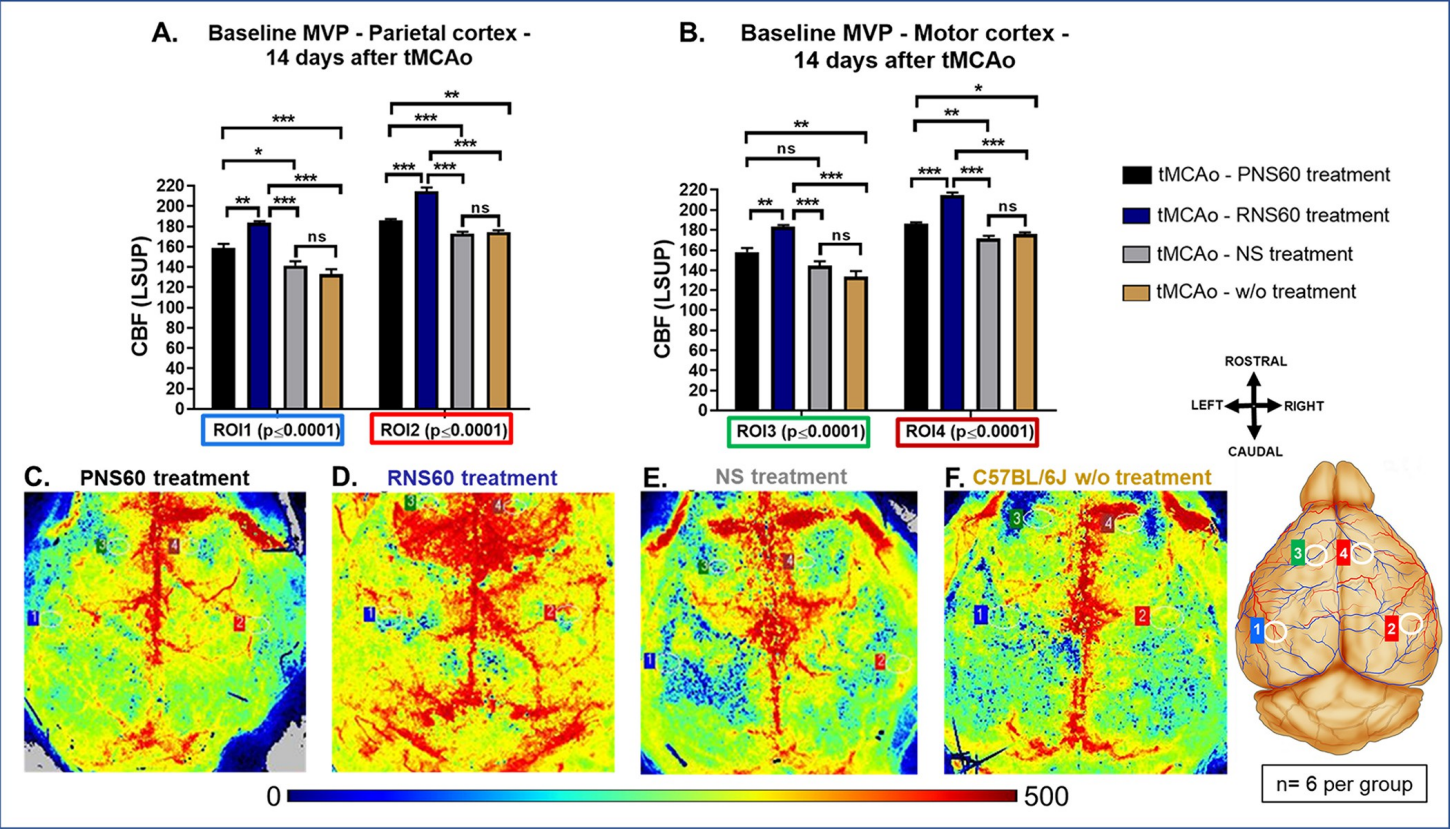
RNS60 treatment increased the cortical cerebral blood flow in mice subjected to tMCAo (60-min) followed by 14 days treatment. Plotted are means ± SEM, showing a significant increase of cerebral cortical microvascular perfusion (MVP) in parietal and motor cortex of both brain sides during baseline period in mice subjected to left transient middle cerebral artery occlusion (tMCAo) treated with PNS60 (black bar), RNS60 (blue bar), NS (grey bar) and without treatment (brown bar) (A and B). Microcirculation (pseudo-colored) images show the cerebral basal blood flow (baseline) of mice subjected to tMCAo followed by 14 days treatment with PNS60 (C), RNS60 (D), NS (E) and without treatment (F). Each image represents an independent mouse. *p<0.05, **p<0.01, ***p<0.001, ns = non-significant.

On day 14, before euthanasia, cortical cerebral MVP was measured in animals subjected to left tMCAo under 3 conditions (a) 3 min baseline, (b) 10 s transient occlusion of the right CCA (to measure % Δ Downfall) and 3 min recovery in 4 regions of interest (ROI), the ROI 1 or left parietal cortex (1LPC); ROI 2 or right parietal cortex (2RPC); ROI 3 or left motor cortex (3LMC), and ROI 4 or right motor cortex (4RMC) (see Methods Section in S2 File and S4F, S4G Fig). Mice treated with RNS60 exhibited a significant increase in baseline MVP in all ROIs (S6 Table in S1 File, Fig 3A and 3B). tMCAo mice treated with RNS60 also showed a trend toward larger values in Δ Downfall and its percentage of change (% Δ Downfall) in all ROIs after a 10 s transient occlusion, however the effect was not statistically significant (see S11 Table in S1 File and S8 Fig). Comparable results to the baseline were observed in the recovery phase (not shown).

### RNS60 treatment reduced alterations in the expression of myelin basic protein (MBP) in the corpus callosum 14 days after tMCAo

**Fig 4:**
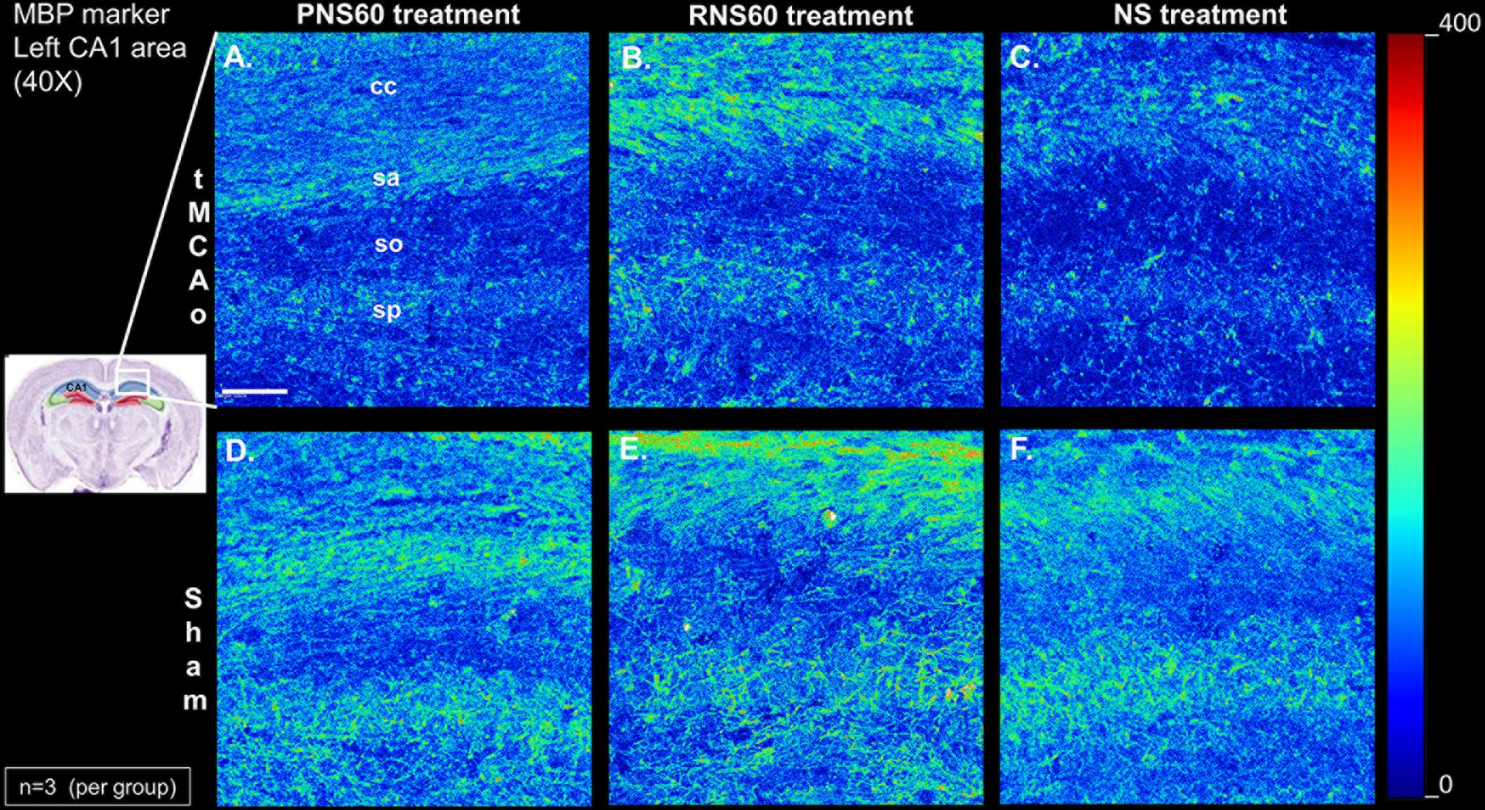
RNS60 treatment of tMCAo mice reduced alterations of myelin basic protein (MBP) in corpus callosum contiguous to the CA1 of the hippocampus. MBP staining in tMCAo mice with RNS60 treatment (B) shows a similar pattern and intensity as in Sham mice (D-F) whereas PNS60- (A) and NS-treated (C) mice showed an altered pattern and lower intensity. A representative staining from each treatment group is shown with colormap intensity scale where red is the highest intensity and blue the lowest. cc, corpus callosum; sa, stratum alveus; so, stratum oriens; sp, stratum pyramidale. Each panel represents an independent mouse (A-C n = 3 and D-F n = 3). Scale bar = 50μm.

We investigated the organization and the intensity levels of MBP in oligodendrocytes. After 14 days of treatment, RNS60 (Fig 4B) caused a reduction in the changes of MBP expression (S7 Table in S1 File), and its pattern of distribution compared with tMCAo mice treated with PNS60 (Fig 4A) or NS (Fig 4C).

### RNS60 treatment reduced neuronal cell loss and extracellular Aß aggregation in the CA3 region of the hippocampus 14 days after tMCAo

**Fig 5:**
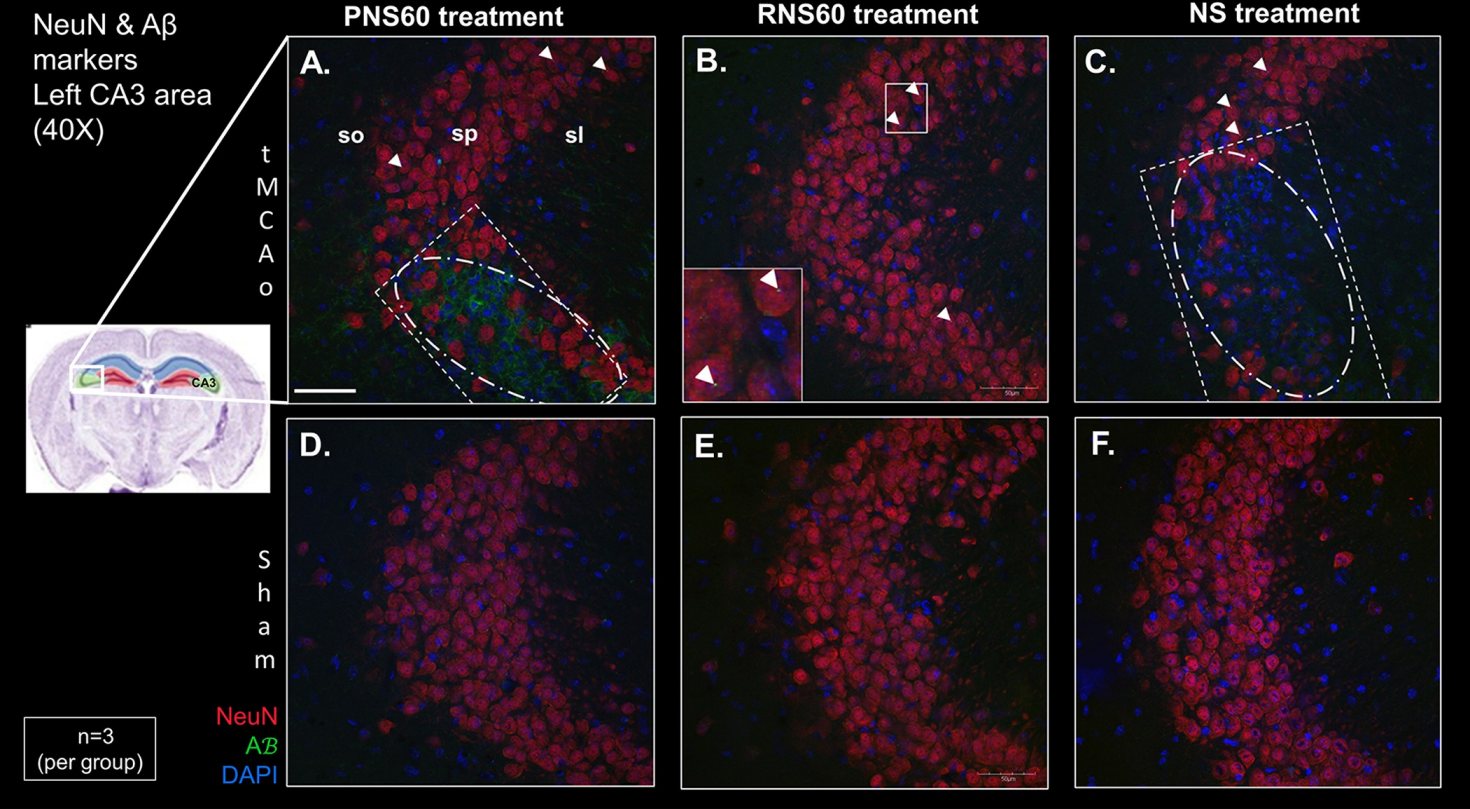
RNS60 treatment decreased Aß aggregation and neurodegeneration in the stratum pyramidale in the CA3 region of the hippocampus of tMCAo animals 14 days after stroke. After 14 days of treatment, mice (n = 3) were sacrificed, and the brains were processed to quantitate Aß and NeuN levels by immunofluorescence. Representative microphotographs of Aß (green), NeuN (red) and DAPI (blue) in the stratum pyramidale (sp) in the CA3 region of the hippocampus are shown. tMCAo (A—C) and Sham (D–F) animals treated with PNS60 (A, D), RNS60 (B, E) and NS (C, F). Rectangle = area of neuronal cell loss; oval = area of extracellular Aß; arrowheads = intracellular Aß. so, stratum oriens in CA3; sp, stratum pyramidale in CA3; sl, stratum lucidum in CA3; sr, stratum radiatum in CA3; Scale bar = 50μm.

Mice with left tMCAo surgery treated with RNS60 (Fig 5B) demonstrate significant protection against neuronal loss (shown by NeuN intensity) compared with tMCAo mice treated with PNS60 (Fig 5A) or NS (Fig 5C) (see neuronal loss in the white dotted rectangle in Fig 5A and 5C, plot in S7A Fig, and NeuN intensity quantification in S8 Table in S1 File). In the areas of neuronal cell death, extracellular β amyloid is observed (dotted oval in Fig 5A and 5C, plot in S7B Fig), being most prominent in the animals treated with PNS60. Sporadic intracellular Aß was observed in tMCAo animals with all treatments (arrowheads; Fig A-C and inset in 5B and S9 Table in S1 File).

### RNS60 treatment decreased microglial activation in the CA3 region of the hippocampus 14 days after tMCAo

**Fig 6:**
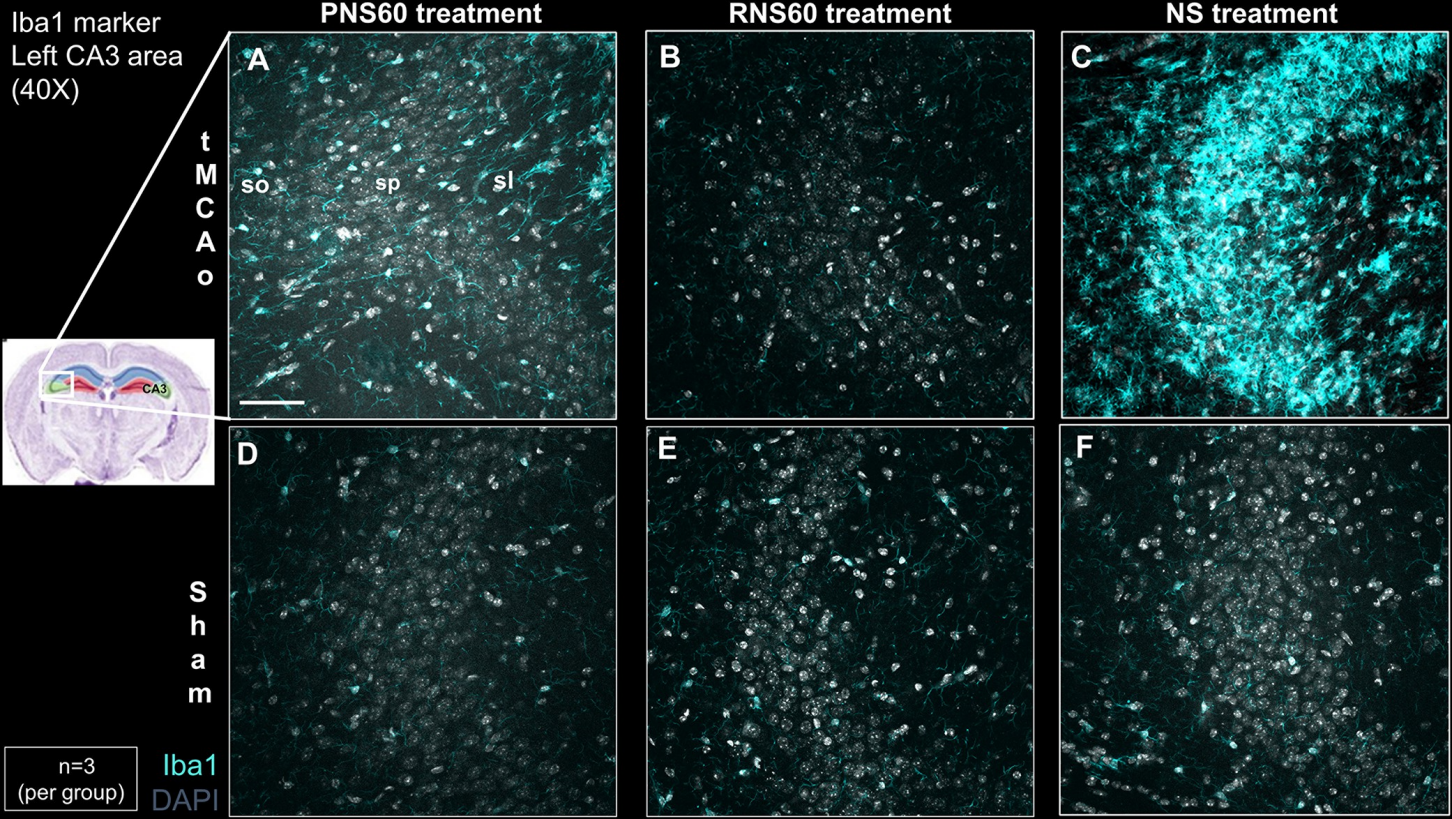
RNS60 treatment of tMCAo mice decreased microglial activation in the CA3 region of the ipsilateral hippocampus. Photomicrographs of Iba1 (light blue) and DAPI (grey) show that tMCAo mice treated with RNS60 (B) exhibit reduced Iba1 signal intensity compared to tMCAo mice treated with PNS60 (A) or NS (C). Iba1 staining in tMCAo mice with RNS60 treatment (B) resembles Sham (D-F). so, stratum oriens; sp, pyramidale stratum; sl, stratum lucidum. Scale bar = 50 μm.

tMCAo mice treated with RNS60 show a decreased microglial activation in the stratum pyramidale of the CA3 region of the ipsilateral hippocampus using Iba1 as a marker (Fig 6). 14 days of RNS60 treatment achieved a significant reduction of microglial activation compared to PNS60 or NS treatments (Fig 6A–6C). tMCAo mice treated with RNS60 (Fig 6B) showed a similar pattern compared to Sham- surgery mice treated with PNS60, RNS60, or NS (Fig 6D–6F). A quantification showed a significant reduction in the intensity of the signal of the Iba1 staining, similar to that obtained in the Sham mice, compared to NS- or PNS60-treated tMCAo mice (S10 Table in S1 File). Triple immunohistochemistry (Iba1, M1 and M2) at day 14 shows that activated microglia (Iba1 positive) do partially overlap with M1 and M2 in tMCAo animals treated with PNS60, but some Iba1 were M1 and M2 negative. In contrast, tMCAo animals treated with NS were M1 negative with a great overlap of Iba1 and M2 whereas the small number of Iba1 positive cells in tMCAo animals treated with RNS60 were M1 and M2 negative (S9 Fig).

### RNS60 treatment decreased hypoxia inducible factor 1α expression four days after unilateral tMCAo

**Fig 7:**
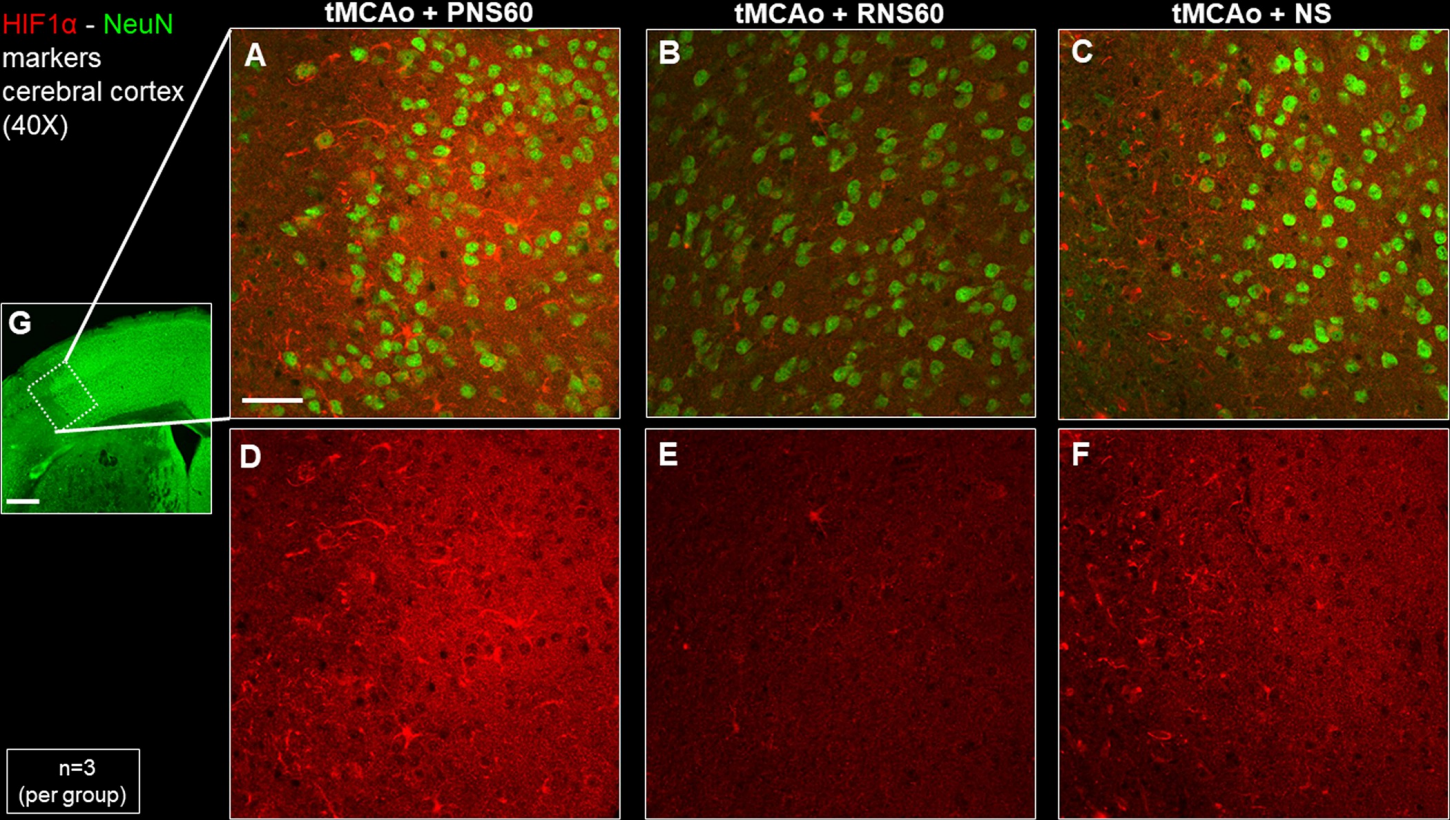
RNS60 treatment decreased expression of hypoxia inducible factor 1α (HIF1α) in the peri infarct cortical area of tMCAo mice 4 days after onset of daily dose of RNS60. Representative photomicrographs of HIF1α (red) and NeuN (green) staining show that tMCAo mice treated with RNS60 (B, E) exhibit reduced HIF1α signal intensity compared to tMCAo mice treated with PNS60 (A, D) or NS (C, F). Inset in G, shows the peri-infarct-penumbra in the cortical area of the brain selected to image A-F. The samples are coronal cerebral slices of the coordinates between 0.62 mm and 0.50 mm anterior to Bregma. Scale bar A-F = 50 μm; and G = 500 μm (n = 3 per group).

With a small cohort of animals, we tested whether RNS60 can decrease hypoxia in the peri-infarct-penumbra. We used the expression of hypoxia inducible factor 1α (HIF1α) as a marker for hypoxia. As shown in Fig 7, RNS60 reduced the expression of HIF1α compared to tMCAo mice treated with PNS60 or NS. The differences between RNS60- and PNS60- or NS-treated mice were statistically significant, whereas HIF1α expression was similar in PNS60 and NS-treated mice (S16 Table in S1 File).

## Discussion

RNS60 is a saline containing oxygen solution without any other active pharmaceutical ingredient. RNS60’s biological activity is most likely due to charge-stabilized nanobubbles [23]. The basic properties of the solution have been described; however, more advanced biophysical technologies are required to further characterize RNS60 [23, 27]. The approach to study biological effects of RNS60 has been by comparative analysis of data from in vitro experiments where signaling pathway of different cell types have been elucidated with data obtained from IP treatment of in vivo animal experiments of different brain pathologies [24, 25, 28, 29, 34, 38–42].

Previously RNS60 has been studied in multiple neurodegenerative disease models including multiple sclerosis, amyotrophic lateral sclerosis, traumatic brain injury, Alzheimer’s disease, and Parkinson’s disease. In these models, RNS60 reduced glial inflammation via suppression of NFkβ and protected neurons via the activation of Akt pro-survival pathway [27–29, 34, 41]. Although this is the first article in which the role of RNS60 in ischemic stroke is investigated, it should be noted that in traumatic brain injury, RNS60 was reported to suppress glial activation, neuronal apoptosis, and memory loss after an acute trauma [27], which is consistent with what we observed. More recently, RNS60 was also reported to increase mitochondrial biogenesis and function in neuronal cells [42], which may play an important role in acute ischemic stroke. Here, we extended the investigation of RNS60 into a mouse model of ischemic stroke to test for its therapeutic effects on infarct size and cognitive damage after ischemic stroke.

To measure tissue damage, two routine histological measurement methods (TTC and H&E) were used in combination with immuno-histological markers of tissue damage. RNS60 treatment led to a reduction in the loss of the compromised brain area, and a reduction in tissue damage reflected in the immuno-histological markers of (a) myelin loss, (b) neuronal death, (c) appearance of Aβ in areas of neuronal death, and (d) activation of microglia.

The prevention of myelin basic protein loss by RNS60 in tMCAo animals is consistent with previous observations (Fig 4). In vitro studies have shown that RNS60 increased maturation and survival of oligodendrocyte precursor cells under metabolic stress, and induced expression of CREB-dependent myelin genes by oligodendrocytes [25, 43]. Our data in tMCAo also agree with data reported by Mondal et al, who demonstrated that RNS60 markedly restored myelin levels in the cerebellum and spinal cord of mice with experimental autoimmune encephalitis, a model of multiple sclerosis [34].

The prevention of neuronal loss by RNS60 in animals with tMCAo is also consistent with previous data. In hippocampal cultured neurons treated with RNS60 show to prevent Aβ -induced apoptosis where the solution decrease Aβ (1–42)- induced tau phosphorylation via (PI-3 kinase—AKT)- mediated inhibition of GSK-β. In tg5XFAD mice, an Alzheimer’s disease model, RNS60 treatment suppress neuronal apoptosis, attenuate tau phosphorylation, inhibit glia activation, reduces Aβ plaques aggregation and protects memory [28]. In our data, it is noteworthy that in animals treated with PNS60 solution, which presented intermediate neuronal loss, an increase in Aβ was observed in the area where cell death occurs, which did not occur in animals treated with NS, which exhibited even greater neuronal loss (Fig 5). The appearance of Aβ can be early and transitional, as a sign or mediator of toxicity activated by ischemia, which triggers cell death and, subsequently is cleared away [44, 45]. It would be appropriate to carry out detailed temporal studies of the progression of ischemic damage, related to the appearance and disappearance of Aβ in the stages after tMCAo, to verify the veracity of this statement. In any case, the data reported in the present study are consistent with the effects of RNS60 in relation to the inhibition of Aβ-mediated neuronal apoptosis, in the Alzheimer’s disease model studied by Modi et al. [28].

In microglia and astrocytes, it has been previously reported that RNS60 suppresses iNOS expression, thereby attenuating glial activation [23]. Simultaneously, in these cells, RNS60 activates the transcription factor CREB through the PI3K1α-Akt pathway. CREB then boosted the transcription of IkBα and inhibited the activation of the transcription factor NF-kB, resulting in an anti-inflammatory effect on microglia. Similar results have been obtained in models of Parkinson’s [29], Alzheimer’s [28], Amyotrophic lateral sclerosis [41] and traumatic brain injury [27]. Based on the fact that inflammatory processes similar to those previously described occur in cerebral ischemia/reperfusion [46–49], in the present investigation the microglial response to treatment with RNS60 was evaluated after stroke, finding an indisputable attenuation of neuroinflammation that becomes evident by the significant decrease in microglial activation in tMCAo mice treated with RNS60, compared to tMCAo mice treated with PNS60 or NS.

Based on the anti-inflammatory effects of RNS60 shown in the prior studies, we expected that Iba1 staining of sections, analyzed on day 14, would overlap with M2 staining, however, that was not the case (S9 Fig). According to a recent review by Benusa et al. [50] the classification of microglia activation into “classic” (M1) and “alternative” (M2) it is an oversimplification that fails to accurately represent array of cellular phenotypes. In the same article it is mentioned that multiple subclasses of microglia do not belong to the M1/M2 classification. Further studies will be needed to characterize microglia subpopulation that are Iba1 (+) M1/M2 (-).

When microvascular perfusion was measured, we observed an increase on the baseline overall circulation (Fig 3 and S6 Table in S1 File) with higher dependency of the contralateral circulation reflected by the trend of larger values of Δ Downfall and % Δ Downfall (S8 Fig and S11 Table in S1 File) suggesting that RNS60 may increase angiogenesis. Further analysis is necessary to explore whether RNS60 treatment activates angiogenesis after stroke.

To measure cognitive performance, NOR and APA tests were used. In the first cognitive task, which is purely a learning and memory task driven by an innate curiosity of mice to explore new objects, the RNS60 solution was effective in completely recovering the animals’ ability to perform this task. Interestingly, the saline solution with added dissolved oxygen alone (PNS60) produced an adverse effect both in the NOR test and the infarct size for reasons that are unclear. However, such negative effects may be attributed to an increase in oxidative stress, and oxygen therapies have failed to provide a therapeutic effect in stroke [51]. Interestingly RNS60 was shown to reduce oxidative stress in a mouse model of ALS [41]. A clear therapeutic effect of RNS60 in reversing or preventing memory damage was also seen in the APA test. In this case, different types of parameters were measured, such as T1stE, which reflects memory and MTA, which reflects learning and functional memory simultaneously. Both were completely recovered with RNS60 treatment. In addition, it is noteworthy that the APA conflict task also showed significant recovery. This test has been associated with the pattern separation process of the hippocampus, which is dependent on neurogenesis in the dentate gyrus [52], suggesting that neurogenesis in the hippocampus is protected (or recovered) by RNS60. It should be noted that adult neurogenesis includes an initial proliferative stage that has a high energy demand [53]. In general, proliferative stages require an appropriate energy source for cell division [54]. RNS60 has been linked to mitochondrial activation, increased ATP levels, and mitochondrial biogenesis [38, 39, 42, 43]. Future experiments may elucidate whether RNS60 recovers pattern separation due to increased mitochondrial activity in addition to protection from tissue damage in ischemic stroke.

We also measured, in a small cohort of animals, whether a brief treatment of RNS60 can affect the expression of HIF1α (Fig 7 and S16 Table in S1 File). The decrease of the expression of HIF1α in animal treated with RNS60 suggests that RNS60 might, directly or indirectly, provide oxygen to the hypoxic tissue preventing further damage. NeuN staining was also dimmer and relatively homogeneous in animals treated with RNS60, whereas in animals treated with PNS60 and NS it showed two neuronal subpopulations (Fig 7). One subpopulation displayed a dimmer staining and similar to the one in RNS60-treated mice, and the other displayed a brighter, more intense staining. One of the initial steps happening during apoptosis is chromatin condensation; perhaps the brighter NeuN-positive neurons observed with the control solutions are going into apoptosis. Further analyses will be needed to test whether the brighter neurons are indeed apoptotic. To our knowledge, this is the first attempt to demonstrate that RNS60 has an effect in hypoxic tissue. Techniques like electron paramagnetic resonance (EPR) imaging, functional magnetic resonance imaging (fMRI) and near-infrared spectroscopy will provide further and more accurate insights [55, 56].

Ischemic stroke is currently treated acutely with therapies that help dissolve or remove the clot [6, 7], followed by rehabilitation therapies that allow recovery from paralysis, difficulty in swallowing and speaking, memory loss, emotional problems, or abnormal sensations of pain, cold, or tingling [57, 58]. So far, there is no neuroprotective treatment to complement the current interventions aimed at reperfusion. The results obtained in this work allow us to argue that RNS60 could be such a treatment, with a viable option for an early treatment that reduces cell death in the ischemic penumbra and that promotes improved recovery during the subacute and chronic phases after an ischemic stroke.

## Supporting information

S1 File Supplemental tables (S1-S16 Tables). (DOCX)

S2 File Supplemental methods (S1 Methods-S5 Methods). (DOCX)

S1 Fig Schematic diagram showing the experimental timeline. (TIF)

S2 Fig Schematic diagram showing the experimental timeline to test whether RNS60 treatment reduces hypoxia. Animals with 1 h tMCAo treated with the different solutions and euthanized 4 days after stroke. The daily injections started one hour after tMCAo, ending the treatment on day 3 and euthanizing the animals on day 4.
(TIF)

S3 Fig Active place avoidance (APA) test protocol performed for 3 days consecutively. Tests were performed on days 11 (trials 1–4), 12 (trials 5–8) and 13 (APA conflict with trials 1–4) after tMCAo surgery. Training on days 11 and 12 assess regular learning (3B) and memory process (3C), whereas the day 13 test evaluates the mental flexibility (APA conflict), a form of cognitive discrimination evaluated by changing the location of the shock zone (3D).
(TIF)

S4 Fig Cerebral microvascular perfusion (MVP) was measured by high resolution laser speckle contrast imaging (HR-LSCI) in motor cortex (MC) and parietal cortex (PC) with real time recording. Representative graphic and images show the LSCI baseline (B-C), during right common carotid artery occlusion (rCCAo) (B-D), and after rCCAo (B-E) in a living mouse subjected to tMCAo followed by 14 days reperfusion. Mouse scalp has been exposed the surface to show the place where the laser (see yellow star in A) was located 1 cm before the bregma anatomical point (see white arrow A). Four circular regions of interest (ROIs) were located in the motor (ROIs 3 and 4) and parietal (ROIs 1 and 2) cortex in each cerebral hemisphere (C, D, E, F and G). The graphic shows the arbitrary laser speckle perfusion units (LSPU) of changes in cerebral blood flood (CBF) through the time in each one of ROIs (B). The colormap scales with colors from blue (0) to red (500) represent the blood flow velocity from lower (0) to higher (500) (C, D and E).
(TIF)

S5 Fig Motor performance is affected 24 h after left tMCAo. The values of the motor performance scores in the mNSS (A), BB (B) and FF (C) were expressed as mean ± SEM. Motor performance is affected 24-h after tMCAo (day 2) compared to 24 h before tMCAo (day 0). The graphs show a trend towards better performance by the RNS60 treated mice (blue bars) compared to mice treated with control solutions, without statistical significance. **p<0.01, ns = non-significant.
(TIF)

S6 Fig tMCAo mice treated with RNS60 had a significantly smaller infarct area. The infarct area (arrows in A-C) was measured in male mice subjected to left tMCAo and treated with PNS60 (A), RNS60 (B) and NS (C). Lineal graph (D) shows that the mice treated with RNS60 (blue line) had a smaller infarct area (mean ± SEM) compared to mice treated with PNS60 (black line) or Normal Saline (grey line). cx, cortex; cc, corpus callosum; cpu; caudate putamen striatum. Scale bar = 1 mm.
(TIF)

S7 Fig RNS60 treatment decreases Aß aggregation and neurodegeneration in the stratum pyramidale in the CA3 region of the hippocampus of tMCAo animals 14 days after stroke. Quantification of NeuN (A) and Aß (B). Mice per group (n = 3). *p<0.05, **p<0.01, ***p<0.001.
(EPS)

S8 Fig Cerebral microvascular perfusion (MVP) trend of larger values of Δ Downfall and % Δ Downfall in tMCAo mice treated with RNS60. Measurements of CBF at day 14, show an increased trend of Δ Downfall and % Δ Downfall in animals treated with RNS60. This trend is more pronounced in ROI 1 and 3 of the Δ Downfall.
(TIF)

S9 Fig Fourteen days after tMCAo surgery mice with RNS60 treatment show a decrease in microglial activation Iba1 (+) in the CA3 region of the ipsilateral hippocampus. These Iba1 (+) cells are M1 and M2 (-). Photomicrographs of Iba1 (light blue), M1 (dark blue), M2 (red) and DAPI (grey) show that tMCAo mice treated with RNS60 (B) exhibit reduced Iba1 signal intensity and no M1 and M2 signal intensity compared to tMCAo mice treated with PNS60 (A) or NS (C). The RNS60 staining resembles sham (D-F). tMCAo mice treated with PNS60 the Iba1 staining has some Iba1/M1 (+) and Iba1/M2 (+), whereas most of the tMCAo animals treated with NS (C) are Iba1/M2 (+). so, stratum oriens; sp, pyramidale stratum; sl, stratum lucidum. Scale bar = 50 μm.
(TIF)

S10 Fig Motor skills are not a factor in behavioral differences observed in tMCAo animals with different treatments. Animals going after tMCAo and treated with the different solutions (RNS60, PNS60 and NS) have no statistically significant differences in distance (A, C, E) and speed (B, D, F) when performing NOR (A, B) or APA (C-F), suggesting that the differences observed in Fig 2 are not due to motor skills differences.
(TIF)
