# Supplementary material for: Neuroprotective effect of the RNS60 in a mouse model of transient focal cerebral ischemia
Source: PLoS One. 2024 Jan 2;19(1):e0295504. doi: 10.1371/journal.pone.0295504 (PMC10760892; doi:10.1371/journal.pone.0295504)
Supplement: S1 File — (DOCX) [file pone.0295504.s001.docx]

**Supplemental Tables**

Supplemental Table 1. ANOVA/Tukey statistical analysis of H&E on day 14 showed that RNS60 treatment reduces the infarct area of tMCAo mice (supplemental Fig 6).

| **Statistic test** | **Fisher (F), _degrees of freedom_**  **and significance (p)** | **Post hoc analyzes and Groups compared with analyzes** | **Adjusted P value (post hoc)** | **Limits Inf & sup**  ${\bar{\boldsymbol{x}}}_{\boldsymbol{i}}\boldsymbol{-}{\bar{\boldsymbol{x}}}_{\boldsymbol{j}}$ **(IC 95%)** |
| --- | --- | --- | --- | --- |
| ANOVA | F_(8, 30)_= 2.00, p˂0.081 interaction | N/A | N/A | N/A |
| ANOVA | F_(4, 30)_= 1.02, p˂0.415  slices | N/A | N/A | N/A |
| ANOVA | F_(2, 30)_= 27.16, p˂0.001  Solution type | Tukey  RNS60/tMCAo – NS/tMCAo | (p˂0.001) | [-3.16, -1.11] |
| ANOVA | F_(2, 30)_= 27.16, p˂0.001  Solution type | Tukey  RNS60/tMCAo – PNS60/tMCAo | (p˂0.001) | [-3.96, -1.91] |
| ANOVA | F_(2, 30)_= 27.16, p˂0.001  Solution type | Tukey  PNS60/tMCAo – NS/tMCAo | (p˂0.148) | [-0.22, 1.82] |

Supplemental Table 2. ANOVA/Tukey statistical analysis of TTC on day 14 showed that RNS60 treatment reduces the hemispheric loss of tMCAo mice (Fig 1).

| **Statistic test** | **Fisher (F), _degrees of freedom_**  **and significance (p)** | **Post hoc analyzes and Groups compared with analyzes** | **Adjusted P value (post hoc)** | **Limits Inf & sup**  ${\bar{\boldsymbol{x}}}_{\boldsymbol{i}}\boldsymbol{-}{\bar{\boldsymbol{x}}}_{\boldsymbol{j}}$ **(IC 95%)** |
| --- | --- | --- | --- | --- |
| ANOVA | F_(3, 12)_= 21.36, p˂0.001 | Tukey  RNS60/tMCAo – PNS60/tMCAo | (p˂0.001) | [12.64, 40.51] |
| ANOVA | F_(3, 12)_= 21.36, p˂0.001 | Tukey  RNS60/tMCAo – NS/tMCAo | (p˂0.002) | [9.52, 37.39] |
| ANOVA | F_(3, 12)_= 21.36, p˂0.001 | Tukey  RNS60/tMCAo –tMCAo w/o Tx | (p˂0.001) | [22.20, 50.08] |
| ANOVA | F_(3, 12)_= 21.36, p˂0.001 | Tukey  PNS60/tMCAo – NS/tMCAo | (p˂0.908) | [-17.06, 10.81] |
| ANOVA | F_(3, 12)_= 21.36, p˂0.001 | Tukey  PNS60/tMCAo –tMCAo w/o Tx | (p˂0.228) | [-4.37, 23.50] |
| ANOVA | F_(3, 12)_= 21.36, p˂0.001 | Tukey  NS/tMCAo –tMCAo w/o Tx | (p˂0.079) | [-1.25, 26.62] |

Supplemental Table 3. Statistic data of NOR test. Statistic information evidencing the effect of RNS60 treatment in tMCAo mice and subjected to NOR on day 7 behave as healthy C57BL/6J mice without surgery and treatment (Fig 2 A)

| **Statistic test** | **Fisher (F), _degrees of freedom_**  **and significance (p)** | **Post hoc analyzes and Groups compared with analyzes** | **Adjusted P value (post hoc)** | **Limits Inf & sup**  ${\bar{\boldsymbol{x}}}_{\boldsymbol{i}}\boldsymbol{-}{\bar{\boldsymbol{x}}}_{\boldsymbol{j}}$ **(IC 95%)** |
| --- | --- | --- | --- | --- |
| ANOVA | F_(3, 30)_= 9.02, p˂0.001 | Tukey  RNS60/tMCAo – PNS60/tMCAo | (p˂0.019) | [0.74, 1.04] |
| ANOVA | F_(3, 30)_= 9.02, p˂0.001 | Tukey  RNS60/tMCAo – NS/tMCAo | (p˂0.001) | [0.29, 1.25] |
| ANOVA | F_(3, 30)_= 9.02, p˂0.001 | Tukey  RNS60/tMCAo – C57BL/6J w/o Sx and Tx | (p˂0.999) | [-0.49, 0.54] |
| ANOVA | F_(3, 30)_= 9.02, p˂0.001 | Tukey  PNS60/tMCAo – NS/tMCAo | (p˂0.615) | [-0.70, 0.26] |
| ANOVA | F_(3, 30)_= 9.02, p˂0.001 | Tukey  PNS60/tMCAo – C57BL/6J w/o Sx and Tx | (p˂0.043) | [-1.04, -0.01] |
| ANOVA | F_(3, 30)_= 9.02, p˂0.001 | Tukey  NS/tMCAo – C57BL/6J w/o Sx and Tx | (p˂0.002) | [-1.25, -0.23] |

Supplemental Table 4. Statistic information evidencing the effect of RNS60 treatment in mice subjected to tMCAo on the maximum time to avoid the shock zone in the APA test. ANOVA/Tukey for days 11 and 12 correspond to graph presented in Figure 2B and ANOVA/Tukey for day 13 corresponds to graph presented in Figure 2D.

| **Statistic test** | **Evaluated Day (D)**  **&**  **Trial (T)** | **Fisher (F), _degrees of freedom_**  **and significance (p)** | **Post hoc analyzes and Groups compared with analyzes** | **Adjusted**  **P value (post hoc)** | **Limits Inf & sup** ${\bar{\boldsymbol{x}}}_{\boldsymbol{i}}\boldsymbol{-}{\bar{\boldsymbol{x}}}_{\boldsymbol{j}}$ **(IC 95%)** |
| --- | --- | --- | --- | --- | --- |
| ANOVA | D11^th^ & T1 | F_(2, 21)_= 7.95, p˂0.003 | Tukey  RNS60 – PNS60 | (p˂0.004) | [33.31, 185.69] |
| ANOVA | D11^th^ & T1 | F_(2, 21)_= 7.95, p˂0.003 | Tukey  RNS60 – NS | (p˂0.010) | [22.18, 174.57] |
| ANOVA | D11^th^ & T1 | F_(2, 21)_= 7.95, p˂0.003 | Tukey  PNS60 – NS | (p˂0.928) | [-87.32, 65.07] |
| ANOVA | D11^th^ & T4 | F_(2, 21)_= 5.63, p˂0.011 | Tukey  RNS60 – PNS60 | (p˂0.037) | [9.36, 333.39] |
| ANOVA | D11^th^ & T4 | F_(2, 21)_= 5.63, p˂0.011 | Tukey  RNS60 – NS | (p˂0.014) | [37.11, 361.14] |
| ANOVA | D11^th^ & T4 | F_(2, 21)_= 5.63, p˂0.011 | Tukey  PNS60 – NS | (p˂0.903) | [-134.27, 189.77] |
| ANOVA | D12^th^ & T8 | F_(2, 21)_= 6.73, p˂0.006 | Tukey  RNS60 – PNS60 | (p˂0.039) | [7.71, 322.79] |
| ANOVA | D12^th^ & T8 | F_(2, 21)_= 6.73, p˂0.006 | Tukey  RNS60 – NS | (p˂0.005) | [62.71, 377.79] |
| ANOVA | D12^th^ & T8 | F_(2, 21)_= 6.73, p˂0.006 | Tukey  PNS60 – NS | (p˂0.658) | [-102.54, 212.54] |
| ANOVA | D13^th^ & T2c | F_(2, 21)_= 4.03, p˂0.033 | Tukey  RNS60 – PNS60 | (p˂0.117) | [-42.15, 262.15] |
| ANOVA | D13^th^ & T2c | F_(2, 21)_= 4.03, p˂0.033 | Tukey  RNS60 – NS | (p˂0.028) | [16.60, 320.90] |
| ANOVA | D13^th^ & T2c | F_(2, 21)_= 4.03, p˂0.033 | Tukey  PNS60 – NS | (p˂0.601) | [-93.40, 210.90] |
| ANOVA | D13^th^ y T4c | F_(2, 21)_= 8.71, p˂0.002 | Tukey  RNS60 – PNS60 | (p˂0.032) | [7.81, 194.44] |
| ANOVA | D13^th^ y T4c | F_(2, 21)_= 8.71, p˂0.002 | Tukey  RNS60 – NS | (p˂0.001) | [58.44, 245.06] |
| ANOVA | D13^th^ y T4c | F_(2, 21)_= 8.71, p˂0.002 | Tukey  PNS60 – NS | (p˂0.375) | [-42.69, 143.94] |

Supplemental Table 5. Statistic information evidencing the effect of RNS60 treatment in mice subjected to tMCAo on the time to the 1^st^ entrance to avoid the shock zone in the APA test. Kruskal-Wallis/Bonferroni for days 11 and 12 correspond to graph presented in Figure 2C and Kruskal-Wallis/Bonferroni for day 13 correspond to graph presented in Figure 2E.

| **Statistic test** | **Evaluated Day (D) &**  **Trial (T)** | **Fisher (F), _degrees of freedom_**  **and significance (p)** | **Post hoc analyzes and Groups compared with analyzes** | **Adjusted P value (post hoc)** | **Limits Inf & sup** ${\bar{\boldsymbol{x}}}_{\boldsymbol{i}}\boldsymbol{-}{\bar{\boldsymbol{x}}}_{\boldsymbol{j}}$ **(IC 95%)** |
| --- | --- | --- | --- | --- | --- |
| Kruskal-Wallis | D11^th^ & T1 | H_(2)_= 1.024, p˂0.599 | Bonferroni  RNS60 – PNS60 | --- | --- |
| Kruskal-Wallis | D11^th^ & T1 | H_(2)_= 1.024, p˂0.599 | Bonferroni  RNS60 –NS | --- | --- |
| Kruskal-Wallis | D11^th^ & T1 | H_(2)_= 1.024, p˂0.599 | Bonferroni  PNS60 – NS | --- | --- |
| Kruskal-Wallis | D11^th^ & T4 | H_(2)_= 11.66, p˂0.003 | Bonferroni  RNS60 – PNS60 | (p˂0.003) | --- |
| Kruskal-Wallis | D11^th^ & T4 | H_(2)_= 11.66, p˂0.003 | Bonferroni  RNS60 – NS | (p˂0.044) | --- |
| Kruskal-Wallis | D11^th^ & T4 | H_(2)_= 11.66, p˂0.003 | Bonferroni  PNS60 – NS | (p˂1.000) | --- |
| Kruskal-Wallis | D12^th^ & T8 | H_(2)_= 11.38, p˂0.003 | Bonferroni  RNS60 – PNS60 | (p˂0.012) | --- |
| Kruskal-Wallis | D12^th^ & T8 | H_(2)_= 11.38, p˂0.003 | Bonferroni  RNS60 – NS | (p˂0.009) | --- |
| Kruskal-Wallis | D12^th^ & T8 | H_(2)_= 11.38, p˂0.003 | Bonferroni  RNS60 – PNS60 | (p˂1.000) | --- |
| Kruskal-Wallis | D13^th^ & T2c | H_(2)_= 9.42, p˂0.009 | Bonferroni  RNS60 – PNS60 | (p˂0.033) | --- |
| Kruskal-Wallis | D13^th^ & T2c | H_(2)_= 9.42, p˂0.009 | Bonferroni  RNS60 – NS | (p˂0.017) | --- |
| Kruskal-Wallis | D13^th^ & T2c | H_(2)_= 9.42, p˂0.009 | Bonferroni  PNS60 – NS | (p˂1.000) | --- |
| Kruskal-Wallis | D13^th^ y T4c | H_(2)_= 9.07, p˂0.011 | Bonferroni  RNS60 – PNS60 | (p˂0.290) | --- |
| Kruskal-Wallis | D13^th^ y T4c | H_(2)_= 9.07, p˂0.011 | Bonferroni  RNS60 – NS | (p˂0.008) | --- |
| Kruskal-Wallis | D13^th^ y T4c | H_(2)_= 9.07, p˂0.011 | Bonferroni  PNS60 – NS | (p˂0.537) | --- |

Supplemental Table 6. Statistic information evidencing the effect of RNS60 treatment in mice subjected to tMCAo on the microvascular perfusion baseline in 4 brain regions of interest (ROI) (Fig 3).

| **Statistic test** | **#ROI and**  **brain region location** | **Fisher (F), _degrees of freedom_**  **and significance (p)** | **Post hoc analyzes and Groups compared with analyzes** | **Adjusted**  **P value**  **(post hoc)** | **Limits inf & sup** ${\bar{\boldsymbol{x}}}_{\boldsymbol{i}}\boldsymbol{-}{\bar{\boldsymbol{x}}}_{\boldsymbol{j}}$ **(IC 95%)** |
| --- | --- | --- | --- | --- | --- |
| ANOVA | ROI1 - CPi | F_(3, 20)_= 32.91, p˂0.001 | Tukey  RNS60 – PNS60 | (p˂0.001) | [9.67, 40.43] |
| ANOVA | ROI1 - CPi | F_(3, 20)_= 32.91, p˂0.001 | Tukey  RNS60 – NS | (p˂0.0001) | [26.68, 57.44] |
| ANOVA | ROI1 - CPi | F_(3, 20)_= 32.91, p˂0.001 | Tukey  RNS60 – w/o Tx | (p˂0.0001) | [35.18, 65.94] |
| ANOVA | ROI1 - CPi | F_(3, 20)_= 32.91, p˂0.001 | Tukey  PNS60 – NS | (p˂0.027) | [1.63, 32.39] |
| ANOVA | ROI1 - CPi | F_(3, 20)_= 32.91, p˂0.001 | Tukey  PNS60 – w/o Tx | (p˂0.001) | [10.13, 40.89] |
| ANOVA | ROI2 - CPd | F_(3, 20)_= 69.57, p˂0.0001 | Tukey  RNS60 – PNS60 | (p˂0.0001) | [19.65, 38.03] |
| ANOVA | ROI2 - CPd | F_(3, 20)_= 69.57, p˂0.0001 | Tukey  RNS60 – NS | (p˂0.0001) | [32.71, 51.09] |
| ANOVA | ROI2 - CPd | F_(3, 20)_= 69.57, p˂0.0001 | Tukey  RNS60 – w/o Tx | (p˂0.0001) | [30.95, 49.33] |
| ANOVA | ROI2 - CPd | F_(3, 20)_= 69.57, p˂0.0001 | Tukey  PNS60 – NS | (p˂0.004) | [3.87, 22.25] |
| ANOVA | ROI2 - CPd | F_(3, 20)_= 69.57, p˂0.0001 | Tukey  PNS60 – w/o Tx | (p˂0.013) | [2.11, 20.49] |
| ANOVA | ROI3 - CMi | F_(3, 20)_= 26.45, p˂0.001 | Tukey  RNS60 – PNS60 | (p˂0.002) | [8.60, 41.65] |
| ANOVA | ROI3 - CMi | F_(3, 20)_= 26.45, p˂0.001 | Tukey  RNS60 – NS | (p˂0.0001) | [22.43, 55.48] |
| ANOVA | ROI3 - CMi | F_(3, 20)_= 26.45, p˂0.001 | Tukey  RNS60 – w/o Tx | (p˂0.0001) | [33.19, 66.24] |
| ANOVA | ROI3 - CMi | F_(3, 20)_= 26.45, p˂0.001 | Tukey  PNS60 – NS | (p˂0.122) | [-2.69, 30.35] |
| ANOVA | ROI3 - CMi | F_(3, 20)_= 26.45, p˂0.001 | Tukey  PNS60 – w/o Tx | (p˂0.002) | [8.07, 41.12] |
| ANOVA | ROI4 - CMd | F_(3, 20)_= 77.29, p˂0.001 | Tukey  RNS60 – PNS60 | (p˂0.0001) | [19.38, 36.87] |
| ANOVA | ROI4 - CMd | F_(3, 20)_= 77.29, p˂0.001 | Tukey  RNS60 – NS | (p˂0.0001) | [34.45, 51.94] |
| ANOVA | ROI4 - CMd | F_(3, 20)_= 77.29, p˂0.001 | Tukey  RNS60 – w/o Tx | (p˂0.0001) | [30.11, 47.60] |
| ANOVA | ROI4 - CMd | F_(3, 20)_= 77.29, p˂0.001 | Tukey  PNS60 – NS | (p˂0.001) | [6.32, 23.82] |
| ANOVA | ROI4 - CMd | F_(3, 20)_= 77.29, p˂0.001 | Tukey  PNS60 – w/o Tx | (p˂0.013) | [1.99, 19.48] |

Supplemental Table 7. Statistic data of MBP marker IF.

| **Statistic test** | **IF marker type** | **Fisher (F), _degrees of freedom_**  **and significance (p)** | **Post hoc analyzes and Groups compared with analyzes** | **Adjusted P value (post hoc)** | **Limits Inf & sup**  ${\bar{\boldsymbol{x}}}_{\boldsymbol{i}}\boldsymbol{-}{\bar{\boldsymbol{x}}}_{\boldsymbol{j}}$ **(IC 95%)** |
| --- | --- | --- | --- | --- | --- |
| ANOVA | MBP | F_(5, 12)_= 72.50, p˂0.001 | Tukey  RNS60/tMCAo – PNS60/tMCAo | (p˂0.001) | [33.49, 89.43] |
| ANOVA | MBP | F_(5, 12)_= 72.50, p˂0.001 | Tukey  RNS60/tMCAo – NS/tMCAo | (p˂0.004) | [12.49, 68.43] |
| ANOVA | MBP | F_(5, 12)_= 72.50, p˂0.001 | Tukey  RNS60/tMCAo – PNS60/Sham | (p˂0.999) | [-31.06, 24.88] |
| ANOVA | MBP | F_(5, 12)_= 72.50, p˂0.001 | Tukey  RNS60/tMCAo – RNS60/Sham | (p˂0.001) | [-101.85, -45.91] |
| ANOVA | MBP | F_(5, 12)_= 72.50, p˂0.001 | Tukey  RNS60/tMCAo – NS/Sham | (p˂0.003) | [-69.60, -13.66] |
| ANOVA | MBP | F_(5, 12)_= 72.50, p˂0.001 | Tukey  PNS60/tMCAo – NS60/tMCAo | (p˂0.192) | [-48.97, 6.97] |
| ANOVA | MBP | F_(5, 12)_= 72.50, p˂0.001 | Tukey  PNS60/tMCAo – PNS60/Sham | (p˂0.001) | [-92.52, -36.58] |
| ANOVA | MBP | F_(5, 12)_= 72.50, p˂0.001 | Tukey  PNS60/tMCAo – RNS60/Sham | (p˂0.001) | [-163.31, -107.37] |
| ANOVA | MBP | F_(5, 12)_= 72.50, p˂0.001 | Tukey  PNS60/tMCAo – NS/Sham | (p˂0.001) | [-131.06, -75.12] |
| ANOVA | MBP | F_(5, 12)_= 72.50, p˂0.001 | Tukey  NS60/tMCAo – PNS60/Sham | (p˂0.002) | [-71.52, -15.58] |
| ANOVA | MBP | F_(5, 12)_= 72.50, p˂0.001 | Tukey  NS60/tMCAo – RNS60/Sham | (p˂0.001) | [-142.31, -86.37] |
| ANOVA | MBP | F_(5, 12)_= 72.50, p˂0.001 | Tukey  NS60/tMCAo – NS/Sham | (p˂0.001) | [-110.06, -54.12] |

Supplemental Table 8. Statistic data of NeuN marker IF (supplemental Fig 7 A).

| **Statistic test** | **IF marker type** | **Fisher (F), _degrees of freedom_**  **and significance (p)** | **Post hoc analyzes and Groups compared with analyzes** | **Adjusted P value (post hoc)** | **Limits Inf & sup**  ${\bar{\boldsymbol{x}}}_{\boldsymbol{i}}\boldsymbol{-}{\bar{\boldsymbol{x}}}_{\boldsymbol{j}}$ **(IC 95%)** |
| --- | --- | --- | --- | --- | --- |
| ANOVA | NeuN | F_(5, 12)_= 80.22, p˂0.001 | Tukey  RNS60/tMCAo – PNS60/tMCAo | (p˂0.001) | [72.59, 268.33] |
| ANOVA | NeuN | F_(5, 12)_= 80.22, p˂0.001 | Tukey  RNS60/tMCAo – NS/tMCAo | (p˂0.001) | [342.57, 538.31] |
| ANOVA | NeuN | F_(5, 12)_= 80.22, p˂0.001 | Tukey  RNS60/tMCAo – PNS60/Sham | (p˂0.735) | [-57.54, 138.20] |
| ANOVA | NeuN | F_(5, 12)_= 80.22, p˂0.001 | Tukey  RNS60/tMCAo – RNS60/Sham | (p˂1.000) | [-92.55, 103.19] |
| ANOVA | NeuN | F_(5, 12)_= 80.22, p˂0.001 | Tukey  RNS60/tMCAo – NS/Sham | (p˂0.547) | [-147.88, 47.87] |
| ANOVA | NeuN | F_(5, 12)_= 80.22, p˂0.001 | Tukey  PNS60/tMCAo – NS60/tMCAo | (p˂0.001) | [397.89, 593.64] |
| ANOVA | NeuN | F_(5, 12)_= 80.22, p˂0.001 | Tukey  PNS60/tMCAo – PNS60/Sham | (p˂0.006) | [-233.32, -37.58] |
| ANOVA | NeuN | F_(5, 12)_= 80.22, p˂0.001 | Tukey  PNS60/tMCAo – RNS60/Sham | (p˂0.001) | [-268.33, -72.58] |
| ANOVA | NeuN | F_(5, 12)_= 80.22, p˂0.001 | Tukey  PNS60/tMCAo – NS/Sham | (p˂0.001) | [-323.66, -127.91] |
| ANOVA | NeuN | F_(5, 12)_= 80.22, p˂0.001 | Tukey  NS60/tMCAo – PNS60/Sham | (p˂0.001) | [-503.30, -307.56] |
| ANOVA | NeuN | F_(5, 12)_= 80.22, p˂0.001 | Tukey  NS60/tMCAo – RNS60/Sham | (p˂0.001) | [-538.31, -342.57] |
| ANOVA | NeuN | F_(5, 12)_= 80.22, p˂0.001 | Tukey  NS60/tMCAo – NS/Sham | (p˂0.001) | [-593.64, -397.89] |

Supplemental Table 9. Statistic data of Aβ marker IF (supplemental Fig 7 B).

| **Statistic test** | **IF marker type** | **Fisher (F), _degrees of freedom_**  **and significance (p)** | **Post hoc analyzes and Groups compared with analyzes** | **Adjusted P value (post hoc)** | **Limits Inf & sup**  ${\bar{\boldsymbol{x}}}_{\boldsymbol{i}}\boldsymbol{-}{\bar{\boldsymbol{x}}}_{\boldsymbol{j}}$ **(IC 95%)** |
| --- | --- | --- | --- | --- | --- |
| ANOVA | Aβ | F_(5, 12)_= 17.10, p˂0.001 | Tukey  RNS60/tMCAo – PNS60/tMCAo | (p˂0.049) | [-74.18, -0.18] |
| ANOVA | Aβ | F_(5, 12)_= 17.10, p˂0.001 | Tukey  RNS60/tMCAo – NS/tMCAo | (p˂0.012) | [-83.46, -9.46] |
| ANOVA | Aβ | F_(5, 12)_= 17.10, p˂0.001 | Tukey  RNS60/tMCAo – PNS60/Sham | (p˂0.773) | [-22.54, 51.46] |
| ANOVA | Aβ | F_(5, 12)_= 17.10, p˂0.001 | Tukey  RNS60/tMCAo – RNS60/Sham | (p˂0.283) | [-12.18, 61.82] |
| ANOVA | Aβ | F_(5, 12)_= 17.10, p˂0.001 | Tukey  RNS60/tMCAo – NS/Sham | (p˂0.155) | [-7.68, 66.32] |
| ANOVA | Aβ | F_(5, 12)_= 17.10, p˂0.001 | Tukey  PNS60/tMCAo – NS60/tMCAo | (p˂0.953) | [-46.28, 27.72] |
| ANOVA | Aβ | F_(5, 12)_= 17.10, p˂0.001 | Tukey  PNS60/tMCAo – PNS60/Sham | (p˂0.005) | [14.65, 88.64] |
| ANOVA | Aβ | F_(5, 12)_= 17.10, p˂0.001 | Tukey  PNS60/tMCAo – RNS60/Sham | (p˂0.001) | [25.00, 99.00] |
| ANOVA | Aβ | F_(5, 12)_= 17.10, p˂0.001 | Tukey  PNS60/tMCAo – NS/Sham | (p˂0.001) | [29.51, 103.50] |
| ANOVA | Aβ | F_(5, 12)_= 17.10, p˂0.001 | Tukey  NS60/tMCAo – PNS60/Sham | (p˂0.001) | [23.92, 97.92] |
| ANOVA | Aβ | F_(5, 12)_= 17.10, p˂0.001 | Tukey  NS60/tMCAo – RNS60/Sham | (p˂0.001) | [34.28, 108.28] |
| ANOVA | Aβ | F_(5, 12)_= 17.10, p˂0.001 | Tukey  NS60/tMCAo – NS/Sham | (p˂0.001) | [38.78, 112.78] |

Supplemental Table 10. Signal intensity values ​​of Iba1 staining in the CA3 region of the ipsilateral hippocampus of mice subjected to tMCAo or Sham surgery, treated with PNS60, RNS60, and NS.

| **Descriptive** | **N** | **Mean** | **Standard error of the mean (SEM)** | **Mín** | **Max** |
| --- | --- | --- | --- | --- | --- |
| Sham - PNS60 | 3 | 18.69 | 0.83 | 17.78 | 20.33 |
| Sham - RNS60 | 3 | 12.45 | 6.99 | 1.00 | 24.19 |
| Sham - NS | 3 | 15.46 | 8.93 | 26.53 | 54.61 |
| tMCAo – PNS60 | 3 | 59.55 | 15.02 | 41.03 | 89.29 |
| tMCAo – RNS60 | 3 | 28.43 | 5.48 | 17.82 | 36.12 |
| tMCAo– NS | 3 | 706.40 | 43.32 | 620.00 | 755.32 |

Supplemental Table 11. Statistical analysis of ΔDownfall and % ΔDownfall in mice subjected to tMCAo on the microvascular perfusion baseline in 4 brain regions of interest (ROI) (supplemental Fig 8).

| **Statistic test** | **ROI** | **Analysis for** | **Fisher (F), _degrees of freedom_**  **and significance (p)** | **Post hoc analyzes and Groups compared with analyzes** | **Adjusted P value (post hoc)** | **Limits Inf & sup** ${\bar{\boldsymbol{x}}}_{\boldsymbol{i}}\boldsymbol{-}{\bar{\boldsymbol{x}}}_{\boldsymbol{j}}$ **(IC 95%)** |
| --- | --- | --- | --- | --- | --- | --- |
| Kruskal-Wallis | 1 | **ΔDownfall** | H_(3)_= 4.582, p˂0.205 | Bonferroni  PNS60 – RNS60 – NS –  w/o TX | --- | --- |
| Kruskal-Wallis | 2 | **ΔDownfall** | H_(3)_= 2.887, p˂0.409 | Bonferroni  PNS60 – RNS60 – NS –  w/o TX | --- | --- |
| Kruskal-Wallis | 3 | **ΔDownfall** | H_(3)_= 6.407, p˂0.093 | Bonferroni  PNS60 – RNS60 – NS –  w/o TX | --- | --- |
| Kruskal-Wallis | 4 | **ΔDownfall** | H_(3)_= 3.387, p˂0.336 | Bonferroni  PNS60 – RNS60 – NS –  w/o TX | --- | --- |
| Kruskal-Wallis | 1 | **% ΔDownfall** | H_(3)_= 3.327, p˂0.344 | Bonferroni  PNS60 – RNS60 – NS –  w/o TX | --- | --- |
| Kruskal-Wallis | 2 | **% ΔDownfall** | H_(3)_= 0.707, p˂0.872 | Bonferroni  PNS60 – RNS60 – NS –  w/o TX | --- | --- |
| Kruskal-Wallis | 3 | **% ΔDownfall** | H_(3)_= 3.240, p˂0.356 | Bonferroni  PNS60 – RNS60 – NS –  w/o TX | --- | --- |
| Kruskal-Wallis | 4 | **% ΔDownfall** | H_(3)_= 0.547, p˂0.909 | Bonferroni  PNS60 – RNS60 – NS –  w/o TX | --- | --- |

Supplemental Table 12. Basic solutions properties.

| **Lot Number** | **O2 Level** | **pH** | **NaCl** | **Conductivity** | **Iron** | **Sterility** | **Endotoxin** |
| --- | --- | --- | --- | --- | --- | --- | --- |
| PNS60-021519 | 56ppm  57ppm  57ppm | 6.0 | 97.9% | 16ms/cm | <2ppm | No Growth | <0.050EU/mL |
| RNS60-011719 | 60ppm  59ppm  54ppm | 6.6 | 97.9% | 16ms/cm | <2ppm | No Growth | <0.050EU/mL |
| NS-121817 | 13ppm  12ppm  12ppm | 5.9 | 97.9% | 16ms/cm | <2ppm | No Growth | <0.050EU/mL |

Supplemental Table 13. Statistical analysis of NOR on days 7, 9, and 10 evidencing the effect in motor skills (distance and speed) in tMCAo mice with different treatments (supplemental Fig 10A-B).

| **Statistic test** | **Evaluated Day (D)**  **&**  **Motor skill evaluated** | **Fisher (F), _degrees of freedom_**  **and significance (p)** | **Post hoc analyzes and Groups compared with analyzes** | **Adjusted**  **P value (post hoc)** | **Limits Inf & sup** ${\bar{\boldsymbol{x}}}_{\boldsymbol{i}}\boldsymbol{-}{\bar{\boldsymbol{x}}}_{\boldsymbol{j}}$ **(IC 95%)** |
| --- | --- | --- | --- | --- | --- |
| ANOVA | D7^th^ & Distance | F_(2, 24)_= 0.125, p˂0.883 | Tukey  RNS60 – PNS60 | (p˂0.921) | [-5.557, 4.069] |
| ANOVA | D7^th^ & Distance | F_(2, 24)_= 0.125, p˂0.883 | Tukey  RNS60 – NS | (p˂0.996) | [-4.652, 4.973] |
| ANOVA | D7^th^ & Distance | F_(2, 24)_= 0.125, p˂0.883 | Tukey  PNS60 – NS | (p˂0.886) | [-3.909, 5.718] |
| ANOVA | D9^th^ & Distance | F_(2, 24)_= 0.142, p˂0.868 | Tukey  RNS60 – PNS60 | (p˂0.926) | [-4.253, 5.750] |
| ANOVA | D9^th^ & Distance | F_(2, 24)_= 0.142, p˂0.868 | Tukey  RNS60 – NS | (p˂0.864) | [-3.969, 6.035] |
| ANOVA | D9^th^ & Distance | F_(2, 24)_= 0.142, p˂0.868 | Tukey  PNS60 – NS | (p˂0.989) | [-4.718, 5.286] |
| Kruskal-Wallis | D10^th^ & Distance | H_(2)_= 0.236, p˂0.889 | Tukey  PNS60 – NS | --- | --- |
| Kruskal-Wallis | D10^th^ & Distance | H_(2)_= 0.236, p˂0.889 | Tukey  RNS60 – PNS60 | --- | --- |
| Kruskal-Wallis | D10^th^ & Distance | H_(2)_= 0.236, p˂0.889 | Tukey  RNS60 – NS | --- | --- |
| ANOVA | D7^th^ & Speed | F_(2, 24)_= 0.217, p˂0.807 | Tukey  RNS60 – PNS60 | (p˂0.852) | [-0.019, 0.012] |
| ANOVA | D7^th^ & Speed | F_(2, 24)_= 0.217, p˂0.807 | Tukey  RNS60 – NS | (p˂0.998) | [-0.015, 0.016] |
| ANOVA | D7^th^ & Speed | F_(2, 24)_= 0.217, p˂0.807 | Tukey  PNS60 – NS | (p˂0.824) | [-0.012, 0.019] |
| ANOVA | D9^th^ & Speed | F_(2, 24)_= 0.289, p˂0.751 | Tukey  RNS60 – PNS60 | (p˂0.933) | [-0.015, 0.019] |
| ANOVA | D9^th^ & Speed | F_(2, 24)_= 0.289, p˂0.751 | Tukey  RNS60 – NS | (p˂0.731) | [-0.012, 0.022] |
| ANOVA | D9^th^ & Speed | F_(2, 24)_= 0.289, p˂0.751 | Tukey  PNS60 – NS | (p˂0.914) | [-0.014, 0.019] |
| Kruskal-Wallis | D10^th^ & Speed | H_(2)_= 0.267, p˂0.875 | Tukey  PNS60 – NS | --- | --- |
| Kruskal-Wallis | D10^th^ & Speed | H_(2)_= 0.267, p˂0.875 | Tukey  RNS60 – PNS60 | --- | --- |
| Kruskal-Wallis | D10^th^ & Speed | H_(2)_= 0.267, p˂0.875 | Tukey  RNS60 – NS | --- | --- |

Supplemental Table 14. Statistic information evidencing the effect of RNS60 treatment in motor skills (distance) mice subjected to tMCAo in the APA test. ANOVA/Tukey & Kruskal-Wallis/Bonferroni for days 11 and 12 correspond to graph (supplemental Fig 10C) and ANOVA/Tukey & Kruskal-Wallis/Bonferroni for day 13 corresponds to graph (supplemental Fig 10E).

| **Statistic test** | **Evaluated Day (D),**  **Trial (T) & Motor skill evaluated** | **Fisher (F), _degrees of freedom_**  **and significance (p)** | **Post hoc analyzes and Groups compared with analyzes** | **Adjusted**  **P value (post hoc)** | **Limits Inf & sup** ${\bar{\boldsymbol{x}}}_{\boldsymbol{i}}\boldsymbol{-}{\bar{\boldsymbol{x}}}_{\boldsymbol{j}}$ **(IC 95%)** |
| --- | --- | --- | --- | --- | --- |
| ANOVA | D11^th^ & T1, Distance | F_(2, 27)_= 0.647, p˂0.532 | Tukey  RNS60 – PNS60 | (p˂0.696) | [-1.260, 2.498] |
| ANOVA | D11^th^ & T1, Distance | F_(2, 27)_= 0.647, p˂0.532 | Tukey  RNS60 – NS | (p˂0.526) | [-1.050, 2.708] |
| ANOVA | D11^th^ & T1, Distance | F_(2, 27)_= 0.647, p˂0.532 | Tukey  PNS60 – NS | (p˂0.959) | [-1.669, 2.089] |
| ANOVA | D11^th^ & T2, Distance | F_(2, 27)_= 0.058, p˂0.944 | Tukey  RNS60 – PNS60 | (p˂0.939) | [-2.179, 1.657] |
| ANOVA | D11^th^ & T2, Distance | F_(2, 27)_= 0.058, p˂0.944 | Tukey  RNS60 – NS | (p˂0.975) | [-2.082, 1.753] |
| ANOVA | D11^th^ & T2, Distance | F_(2, 27)_= 0.058, p˂0.944 | Tukey  PNS60 – NS | (p˂0.992) | [-1.822, 2.014] |
| ANOVA | D11^th^ & T3, Distance | F_(2, 27)_= 0.358 p˂0.703 | Tukey  RNS60 – PNS60 | (p˂0.822) | [-2.181, 1.333] |
| ANOVA | D11^th^ & T3, Distance | F_(2, 27)_= 0.358 p˂0.703 | Tukey  RNS60 – NS | (p˂0.974) | [-1.602, 1.912] |
| ANOVA | D11^th^ & T3, Distance | F_(2, 27)_= 0.358 p˂0.703 | Tukey  PNS60 – NS | (p˂0.696) | [-1.178, 2.336] |
| ANOVA | D11^th^ & T4, Distance | F_(2, 27)_= 0.691 p˂0.510 | Tukey  RNS60 – PNS60 | (p˂0.639) | [-2.599, 1.203] |
| ANOVA | D11^th^ & T4, Distance | F_(2, 27)_= 0.691 p˂0.510 | Tukey  RNS60 – NS | (p˂0.980) | [-1.756, 2.046] |
| ANOVA | D11^th^ & T4, Distance | F_(2, 27)_= 0.691 p˂0.510 | Tukey  PNS60 – NS | (p˂0.523) | [-1.058, 2.744] |
| ANOVA | D12^th^ & T5, Distance | F_(2, 27)_= 0.820 p˂0.451 | Tukey  RNS60 – PNS60 | (p˂0.419) | [-2.630, 0.840] |
| ANOVA | D12^th^ & T5, Distance | F_(2, 27)_= 0.820 p˂0.451 | Tukey  RNS60 – NS | (p˂0.832) | [-2.141, 1.329] |
| ANOVA | D12^th^ & T5, Distance | F_(2, 27)_= 0.820 p˂0.451 | Tukey  PNS60 – NS | (p˂0.766) | [-1.246, 2.224] |
| ANOVA | D12^th^ & T6, Distance | F_(2, 27)_= 0.025 p˂0.975 | Tukey  RNS60 – PNS60 | (p˂0.995) | [-1.355, 1.465] |
| ANOVA | D12^th^ & T6, Distance | F_(2, 27)_= 0.025 p˂0.975 | Tukey  RNS60 – NS | (p˂0.973) | [-1.283, 1.537] |
| ANOVA | D12^th^ & T6, Distance | F_(2, 27)_= 0.025 p˂0.975 | Tukey  PNS60 – NS | (p˂0.991) | [-1.338, 1.482] |
| ANOVA | D12^th^ & T7, Distance | F_(2, 27)_= 0.439 p˂0.649 | Tukey  RNS60 – PNS60 | (p˂0.903) | [-1.644, 2.334] |
| ANOVA | D12^th^ & T7, Distance | F_(2, 27)_= 0.439 p˂0.649 | Tukey  RNS60 – NS | (p˂0.623) | [-1.238, 2.274] |
| ANOVA | D12^th^ & T7, Distance | F_(2, 27)_= 0.439 p˂0.649 | Tukey  PNS60 – NS | (p˂0.869) | [-1.583, 2.395] |
| Kruskal-Wallis | D12^th^ & T8, Distance | H_(2)_= 0.266, p˂0.876 | Tukey  PNS60 – NS | --- | --- |
| Kruskal-Wallis | D12^th^ & T8, Distance | H_(2)_= 0.266, p˂0.876 | Tukey  RNS60 – PNS60 | --- | --- |
| Kruskal-Wallis | D12^th^ & T8, Distance | H_(2)_= 0.266, p˂0.876 | Tukey  RNS60 – NS | --- | --- |
| ANOVA | D13^th^ & T1c, Distance | F_(2, 27)_= 0.439 p˂0.649 | Tukey  RNS60 – PNS60 | (p˂0.903) | [-1.644, 2.334] |
| ANOVA | D13^th^ & T1c, Distance | F_(2, 27)_= 0.439 p˂0.649 | Tukey  RNS60 – NS | (p˂0.623) | [-1.238, 2.274] |
| ANOVA | D13^th^ & T1c, Distance | F_(2, 27)_= 0.439 p˂0.649 | Tukey  PNS60 – NS | (p˂0.869) | [-1.583, 2.395] |
| ANOVA | D13^th^ & T2c, Distance | F_(2, 27)_= 0.842 p˂0.442 | Tukey  RNS60 – PNS60 | (p˂0.600) | [-0.931, 2.129] |
| ANOVA | D13^th^ & T2c, Distance | F_(2, 27)_= 0.842 p˂0.442 | Tukey  RNS60 – NS | (p˂0.440) | [-0.765, 2.295] |
| ANOVA | D13^th^ & T2c, Distance | F_(2, 27)_= 0.842 p˂0.442 | Tukey  PNS60 – NS | (p˂0.959) | [-1.323, 1.655] |
| ANOVA | D13^th^ & T3c, Distance | F_(2, 27)_= 2.921 p˂0.072 | Tukey  RNS60 – PNS60 | (p˂0.058) | [-0.304, 2.098] |
| ANOVA | D13^th^ & T3c, Distance | F_(2, 27)_= 2.921 p˂0.072 | Tukey  RNS60 – NS | (p˂0.486) | [-0.566, 1.562] |
| ANOVA | D13^th^ & T3c, Distance | F_(2, 27)_= 2.921 p˂0.072 | Tukey  PNS60 – NS | (p˂0.416) | [-1.572, 0.500] |
| Kruskal-Wallis | D13^th^ & T4c, Distance | H_(2)_= 3.518, p˂0.172 | Tukey  PNS60 – NS | --- | --- |
| Kruskal-Wallis | D13^th^ & T4c, Distance | H_(2)_= 3.518, p˂0.172 | Tukey  RNS60 – PNS60 | --- | --- |
| Kruskal-Wallis | D13^th^ & T4c, Distance | H_(2)_= 3.518, p˂0.172 | Tukey  RNS60 – NS | --- | --- |

Supplemental Table 15. Statistic information evidencing the effect of RNS60 treatment in motor skill (speed) mice subjected to tMCAo in the APA test. ANOVA/Tukey & Kruskal-Wallis/Bonferroni for days 11 and 12 correspond to graph (supplemental Fig 10D) and ANOVA/Tukey & Kruskal-Wallis/Bonferroni for day 13 corresponds to graph (supplemental Fig 10F).

| **Statistic test** | **Evaluated Day (D),**  **Trial (T) & Motor skill evaluated** | **Fisher (F), _degrees of freedom_**  **and significance (p)** | **Post hoc analyzes and Groups compared with analyzes** | **Adjusted**  **P value (post hoc)** | **Limits Inf & sup** ${\bar{\boldsymbol{x}}}_{\boldsymbol{i}}\boldsymbol{-}{\bar{\boldsymbol{x}}}_{\boldsymbol{j}}$ **(IC 95%)** |
| --- | --- | --- | --- | --- | --- |
| ANOVA | D11^th^ & T1, Speed | F_(2, 27)_= 0.513, p˂0.604 | Tukey  RNS60 – PNS60 | (p˂0.976) | [-0.268, 0.318] |
| ANOVA | D11^th^ & T1, Speed | F_(2, 27)_= 0.513, p˂0.604 | Tukey  RNS60 – NS | (p˂0.605) | [-0.179, 0.407] |
| ANOVA | D11^th^ & T1, Speed | F_(2, 27)_= 0.513, p˂0.604 | Tukey  PNS60 – NS | (p˂0.735) | [-0.204, 0.382] |
| ANOVA | D11^th^ & T2, Speed | F_(2, 27)_= 0.003, p˂0.997 | Tukey  RNS60 – PNS60 | (p˂0.996) | [-0.319, 0.341] |
| ANOVA | D11^th^ & T2, Speed | F_(2, 27)_= 0.003, p˂0.997 | Tukey  RNS60 – NS | (p˂1.000) | [-0.327, 0.335] |
| ANOVA | D11^th^ & T2, Speed | F_(2, 27)_= 0.003, p˂0.997 | Tukey  PNS60 – NS | (p˂0.998) | [-0.338, 0.324] |
| ANOVA | D11^th^ & T3, Speed | F_(2, 27)_= 0.191 p˂0.827 | Tukey  RNS60 – PNS60 | (p˂0.919) | [-0.344, 0.249] |
| ANOVA | D11^th^ & T3, Speed | F_(2, 27)_= 0.191 p˂0.827 | Tukey  RNS60 – NS | (p˂0.974) | [-0.271, 0.323] |
| ANOVA | D11^th^ & T3, Speed | F_(2, 27)_= 0.191 p˂0.827 | Tukey  PNS60 – NS | (p˂0.816) | [-0.224, 0.369] |
| ANOVA | D11^th^ & T4, Speed | F_(2, 27)_= 0.278 p˂0.759 | Tukey  RNS60 – PNS60 | (p˂0.837) | [-0.391, 0.245] |
| ANOVA | D11^th^ & T4, Speed | F_(2, 27)_= 0.278 p˂0.759 | Tukey  RNS60 – NS | (p˂0.990) | [-0.301, 0.335] |
| ANOVA | D11^th^ & T4, Speed | F_(2, 27)_= 0.278 p˂0.759 | Tukey  PNS60 – NS | (p˂0.764) | [-0.228, 0.408] |
| ANOVA | D12^th^ & T5, Speed | F_(2, 27)_= 0.129 p˂0.880 | Tukey  RNS60 – PNS60 | (p˂0.870) | [-0.391, 0.259] |
| ANOVA | D12^th^ & T5, Speed | F_(2, 27)_= 0.129 p˂0.880 | Tukey  RNS60 – NS | (p˂0.979) | [-0.351, 0.299] |
| ANOVA | D12^th^ & T5, Speed | F_(2, 27)_= 0.129 p˂0.880 | Tukey  PNS60 – NS | (p˂0.950) | [-0.285, 0.365] |
| ANOVA | D12^th^ & T6, Speed | F_(2, 27)_= 0.135 p˂0.874 | Tukey  RNS60 – PNS60 | (p˂0.871) | [-0.193, 0.291] |
| ANOVA | D12^th^ & T6, Speed | F_(2, 27)_= 0.135 p˂0.874 | Tukey  RNS60 – NS | (p˂0.990) | [-0.229, 0.255] |
| ANOVA | D12^th^ & T6, Speed | F_(2, 27)_= 0.135 p˂0.874 | Tukey  PNS60 – NS | (p˂0.928) | [-0.278, 0.206] |
| ANOVA | D12^th^ & T7, Speed | F_(2, 27)_= 0.856 p˂0.436 | Tukey  RNS60 – PNS60 | (p˂0.868) | [-0.245, 0.371] |
| ANOVA | D12^th^ & T7, Speed | F_(2, 27)_= 0.856 p˂0.436 | Tukey  RNS60 – NS | (p˂0.409) | [-0.147, 0.469] |
| ANOVA | D12^th^ & T7, Speed | F_(2, 27)_= 0.856 p˂0.436 | Tukey  PNS60 – NS | (p˂0.712) | [-0.209, 0.406] |
| Kruskal-Wallis | D12^th^ & T8, Speed | H_(2)_= 1.338, p˂0.512 | Tukey  PNS60 – NS | --- | --- |
| Kruskal-Wallis | D12^th^ & T8, Speed | H_(2)_= 1.338, p˂0.512 | Tukey  RNS60 – PNS60 | --- | --- |
| Kruskal-Wallis | D12^th^ & T8, Speed | H_(2)_= 1.338, p˂0.512 | Tukey  RNS60 – NS | --- | --- |
| ANOVA | D13^th^ & T1c, Speed | F_(2, 27)_= 0.003 p˂0.997 | Tukey  RNS60 – PNS60 | (p˂0.998) | [-0.292, 0.306] |
| ANOVA | D13^th^ & T1c, Speed | F_(2, 27)_= 0.003 p˂0.997 | Tukey  RNS60 – NS | (p˂0.997) | [-0.290, 0.308] |
| ANOVA | D13^th^ & T1c, Speed | F_(2, 27)_= 0.003 p˂0.997 | Tukey  PNS60 – NS | (p˂1.000) | [-0.297, 0.301] |
| ANOVA | D13^th^ & T2c, Speed | F_(2, 27)_= 1.226 p˂0.309 | Tukey  RNS60 – PNS60 | (p˂0.733) | [-0.159, 0.299] |
| ANOVA | D13^th^ & T2c, Speed | F_(2, 27)_= 1.226 p˂0.309 | Tukey  RNS60 – NS | (p˂0.278) | [-0.085, 0.375] |
| ANOVA | D13^th^ & T2c, Speed | F_(2, 27)_= 1.226 p˂0.309 | Tukey  PNS60 – NS | (p˂0.700) | [-0.155, 0.305] |
| ANOVA | D13^th^ & T3c, Speed | F_(2, 27)_= 1.346 p˂0.277 | Tukey  RNS60 – PNS60 | (p˂0.267) | [-0.071, 0.323] |
| ANOVA | D13^th^ & T3c, Speed | F_(2, 27)_= 1.346 p˂0.277 | Tukey  RNS60 – NS | (p˂0.899) | [-0.162, 0.232] |
| ANOVA | D13^th^ & T3c, Speed | F_(2, 27)_= 1.346 p˂0.277 | Tukey  PNS60 – NS | (p˂0.494) | [-0.288, 0.106] |
| Kruskal-Wallis | D13^th^ & T4c, Speed | H_(2)_= 2.739, p˂0.254 | Tukey  PNS60 – NS | --- | --- |
| Kruskal-Wallis | D13^th^ & T4c, Speed | H_(2)_= 2.739, p˂0.254 | Tukey  RNS60 – PNS60 | --- | --- |
| Kruskal-Wallis | D13^th^ & T4c, Speed | H_(2)_= 2.739, p˂0.254 | Tukey  RNS60 – NS | --- | --- |

Supplemental Table 16. Statistic data of HIF1α marker IF.

| **Statistic test** | **IF marker type** | **Fisher (F), _degrees of freedom_**  **and significance (p)** | **Post hoc analyzes and Groups compared with analyzes** | **Adjusted P value (post hoc)** | **Limits Inf & sup**  **(IC 95%)** |
| --- | --- | --- | --- | --- | --- |
| ANOVA | HIF1α | F_(2, 6)_= 135.77, p˂0.001 | Tukey  RNS60/tMCAo – PNS60/tMCAo | (p˂0.001) | [-320.65, -216.54] |
| ANOVA | HIF1α | F_(2, 6)_= 135.77, p˂0.001 | Tukey  RNS60/tMCAo – NS/tMCAo | (p˂0.001) | [-253.51, -149.40] |
| ANOVA | HIF1α | F_(2, 6)_= 135.77, p˂0.001 | Tukey  PNS60/tMCAo – NS60/tMCAo | (p˂0.018) | [15.09, 119.20] |
