# Supplementary material for: Neuroprotective effect of the RNS60 in a mouse model of transient focal cerebral ischemia
Source: PLoS One. 2024 Jan 2;19(1):e0295504. doi: 10.1371/journal.pone.0295504 (PMC10760892; doi:10.1371/journal.pone.0295504)
Supplement: S2 File — (DOCX) [file pone.0295504.s002.docx]

**Supplemental Methods**

**1. Solutions preparation**

It has been previously described by Khasnavis et al (1). In brief, (Tacoma, WA) using a rotor/stator device, which incorporates controlled turbulence and TCP flow. Briefly, sodium chloride (0.9%) for irrigation, USP (pH 5.6) (4.5–7.0, Hospira), was processed at 4 °C, and a flow rate of 32 ml/s under 1 atm of oxygen back-pressure (7.8 ml/s gas flow rate) while maintaining a rotor speed of 3.450 rpm. The resulting fluid was immediately placed into glass bottles (KG-33 borosilicate glass, Kimble-Chase) and sealed using gray chlorobutyl rubber stoppers (USP class 6, West Pharmaceuticals) to maintain pressure and minimize leachables. When tested after 24 h, the oxygen content was 55 +/- 5 ppm. Chemically, RNS60 contains water, sodium chloride, and 50–60 ppm oxygen but no active pharmaceutical ingredients. The controls for RNS60 were also used in this study: (*a*) normal saline (NS) from the same manufacturing batch. This saline contacted the same device surfaces as RNS60 and was bottled in the same way; (*b*) PNS60, saline with the same oxygen content (55 +/- 5 ppm) that was prepared inside the same device but was not processed with TCP flow. Careful analysis demonstrated that all three fluids were chemically identical. The solutions are kept at 4^0^C until injection. IP injections were performed at room temperature.

**2. Basic characterization of RNS60 and control solutions**

A basic characterization of the solutions was previously described (1). Inductively Coupled Plasma Mass spectrometry (ICP-MS) testing for 26 metals and total organic carbon analysis indicated no differences between RNS60 and the control solutions within detection limits. In addition, Liquid Chromatography Quadrupole Time-of-flight Mass spectroscopy (MS-TOF) analysis with and without chromatographic pretreatment, using negative or positive ion models, did not reveal differences. Atomic Force Microscopy was used to compare the size of the bubbles showing differences in size. In addition, time-of-flight mass spectrometry (LC-Q-TOF) revealed no differences between the solutions (2). Supplemental table 12 contains the basic solutions properties used in this article.

**3. Stroke validation**

To validate the stroke, we used (a) functional and (b) structural stroke validation.

(a) Functional stroke validation. Previously described (3), subdivided into (i) modified Neurological Severity Score (mNSS), (ii) Beam Balance (BB), and (iii) Foot Fault (FF). The tests were performed 24 h before and 24 h after surgery.

(i) mNSS: Consists of a neurological motor test, subdivided into:

- Motor test (A) Mouse suspended by tail (with the score from 0-3):

(0) normal response-balance suspension

(1) Inability to suspend contralateral forelimb

(2) Inability to suspend contralateral hindlimb

(3) Head moves >10 degrees off vertical axis within 30 s

- Motor test (B) Place mouse on floor (with the score from 0-3):

(0) normal response-straight walk

(1) Inability to walk straight

(2) Circling toward paretic - ipsilateral side

(3) Falls down to paretic – ipsilateral side

(ii) BB: Mice were placed on a long 1 inch-width beam for 60 s (with the score from 0-6):

(0) normal response is balance with steady posture for >60 s.

(1) grasps side of the beam

(2) hugs beam and 1 limb falls down from beam

(3) hugs beam and 2 limb falls down from beam, or spins

(4) attempts to balance on beam but falls off from 40-59 s

(5) attempts to balance on beam but falls off from 20-39 s

(6) falls off; no attempts to balance or hang on beam in <20 s

(iii) FF: Mice were tested for forelimb movement dysfunction while walking on elevated metal grids with randomly missing support bars. With each weight-bearing step, the forelimb can fall or slip between the metal support bars, which was recorded as a foot fault. The total number of forelimb steps and the total number of foot faults were recorded. The percentage of forelimb foot faults to total steps that occurred within 2 min was calculated.

(b) Structural stroke validation. Subdivided into (i) Hematoxylin & Eosin (H&E), and (ii) 2,3,5-triphenyltetrazolium chloride (TTC) were performed.

(i) Hematoxylin & Eosin (H&E) staining. The area of the stroke was measured using H&E staining and assessed for hemispheric brain loss according to the instructions Abcam (cat# ab245880). After perfusion and fixation, brains were removed, frozen, and sectioned (30 µm). Five sections of the compromised area per animal were taken to measure the size of the stroke. Image acquisition was performed with Aperio ImageScope – pathology slice viewing software (Leica Biosystems) and analyzed with ImageJ software as described (4).

(ii) 2,3,5-triphenyltetrazolium chloride (TTC) staining. TTC staining was performed on day 14. Mouse brains were removed and sectioned using a brain matrix (six coronal sections, 1 mm each/brain). Sections were then immersed in 2% TTC at 37°C for 5-min, scanned and analyzed with ImageJ. Data analysis was performed as described (5).

**4. Perfusion and Fixation**

The animals were perfused through the apex in the left ventricle of the heart for 3-min with 10 mM phosphate buffered saline pH 7.4 (PBS) followed by 4% paraformaldehyde (PFA) in PBS. The brains were removed and post-fixed (48 h) in 4% PFA in PBS and transferred to 30% sucrose in PBS until the brains were isopycnic with the solution. The brains were then quickly frozen in n-methylbutane, cooled over dry ice, and kept at -80oC for cryostat sectioning (6). Free-floating coronal brain sections (30 µm) were obtained with coordinates between 0.62 mm & 0.50 mm anterior to Bregma for the HIF1-α experiment and between -1.34 mm & -1.42 mm posterior to Bregma (7) and processed for H&E and immunofluorescence.

**5. Immunofluorescence**

Materials: Anti-MBP polyclonal chicken antibody ((PA1-10008) Invitrogen, Waltham, USA), anti-NeuN polyclonal rabbit antibody ((Ab104225) Abcam, Cambridge, USA)), anti-β-Amyloid 1-16 (anti-Aβ 6E10) monoclonal mouse antibody ((SIG-39300) Biolegend, San Diego, USA), anti-Iba1 polyclonal rabbit antibody ((019-19741) Wako-Fujifilm, Osaka, Japan), anti-CD16/CD32 monoclonal rat antibody ((14-0161-82) Invitrogen, Waltham, USA), anti-Mannose receptor monoclonal mouse antibody ((Ab8918) Abcam, Cambridge, USA)), anti-HIF1-α polyclonal rabbit antibody ((NB100-134) Novus Biologicals, Colorado, USA)), anti-NeuN polyclonal chicken antibody ((ABN91) Milipore Sigma, Burlington, USA)), Goat anti-Chicken IgY H&L (Alexa Fluor® 488) ((ab150169) Abcam, Cambridge, USA), Goat anti-Rabbit IgG H&L (Alexa Fluor® 568) ((ab175471) Abcam, Cambridge, USA), Goat anti-Mouse IgG H&L (Alexa Fluor® 488) ((ab150113) Abcam, Cambridge, USA), Goat anti-Chicken IgY H&L (Alexa Fluor® 647 preadsorbed) ((ab150175) Abcam, Cambridge, USA), Goat anti-Rat IgG H&L (Alexa Fluor® 647 preadsorbed) ((ab150167) Abcam, Cambridge, USA). VECTASHIELD® HardSet™ Antifade Mounting Medium with DAPI ((H-1500-10) Vector laboratories, Burlington, Canada)). Goat F(ab) anti-mouse (IgG H&L (ab6668) Abcam, Cambridge, USA) was used to quench endogenous IgG.

Quenching of autofluorescence: To reduce autofluorescence, sections were washed 3X (10-min) in PBS and treated (30-min) with glycine solution (PBS - 1% Glycine). To reduce background, sections were rinsed (10-min) with sodium borohydride solution (0.1%) in PBS.

Permeabilization and Immunofluorescence: For figure 7, sections were permeabilized (10-min) with 1% SDS (PBS-Tb) and incubated (1-h) in blocker solution (10% NGS – PBS – 0.1% Triton x-100 (PBS-Tc)). For all the other immunofluorescences sections were permeabilized (15-min) with PBS - 0.25% Triton (PBS-Tb) and incubated (1-h) in blocker solution (10% NGS – PBS – 0.1% Triton x-100 (PBS-Tc)). To quench endogenous nonspecific IgG, brain slices were incubated (2-h) with Goat F(ab) anti-mouse (1:1000) diluted in blocker solution. After quenching, sections were washed 3X (10-min) with PBS and incubated on a rotator at 4°C overnight with primary anti-NeuN (1:500), anti-MBP (1:2000), anti-Iba1 (1:500), anti-Aβ6E10 (1:500), anti-CD16/CD32 (1:50), anti-Mannose receptor (1:100) antibodies diluted in primary antibody solution (10 mM glycine – 0.05% Tween20 – 10% NGS – PBS-Tc). After 12-h, 3X washes were performed with PBS-Tc and incubated (2-h) in the dark with the secondary antibodies including Alexa Fluor® 488 conjugated Goat anti-mouse (1:200), Alexa Fluor® 568 Goat anti-rabbit (1:1000), Alexa Fluor® 647 Goat anti-rat (1:300) and Alexa Fluor® 647-conjugated Goat anti-chicken (1:200). Sections were washed 2X (10- min) with PBS-Tc and 3X (10-min) with PBS and mounted on glass slides with mounting medium with DAPI. Slides were dried at room temperature in the dark for 12-h, and then kept in the freezer at -20°C for at least 12-h more before image acquisition by were determined using the confocal microscopy.

Quantifications: The sections were photographed at 40X magnification, and the images were used to evaluate the fluorescence intensity of MBP immunostaining in ROIs located in the corpus callosum and stratum alveus (above to CA1), and for βA NeuN and Iba1 the chosen ROI for quantification was CA3. Images were obtained using the laser Olympus FV 1000 D microscope and IF was measured with Fluoview FV1000 software (8).

**Supplemental References**

1. Khasnavis S, Jana A, Roy A, Mazumder M, Bhushan B, Wood T, et al. Suppression of nuclear factor-κB activation and inflammation in microglia by physically modified saline. J Biol Chem. 2012;287(35):29529-42.

2. Mondal S, Martinson JA, Ghosh S, Watson R, Pahan K. Protection of Tregs, suppression of Th1 and Th17 cells, and amelioration of experimental allergic encephalomyelitis by a physically-modified saline. PLoS One. 2012;7(12):e51869.

3. Zhou J, Li J, Rosenbaum DM, Barone FC. Thrombopoietin protects the brain and improves sensorimotor functions: reduction of stroke-induced MMP-9 upregulation and blood-brain barrier injury. J Cereb Blood Flow Metab. 2011;31(3):924-33.

4. Nguyen D. Quantifying chromogen intensity in immunohistochemistry via reciprocal intensity. 2013.

5. Cheng J, Shen W, Jin L, Pan J, Zhou Y, Pan G, et al. Treadmill exercise promotes neurogenesis and myelin repair via upregulating Wnt/β‑catenin signaling pathways in the juvenile brain following focal cerebral ischemia/reperfusion. Int J Mol Med. 2020;45(5):1447-63.

6. Hernández AI, Oxberry WC, Crary JF, Mirra SS, Sacktor TC. Cellular and subcellular localization of PKMζ. Philos Trans R Soc Lond B Biol Sci. 2014;369(1633):20130140.

7. Paxinos G, Franklin KB. The Mouse Brain in Stereotaxic Coordinates. Third. Academic Press; 2008.

8. Laing KK, Simoes S, Baena-Caldas GP, Lao PJ, Kothiya M, Igwe KC, et al. Cerebrovascular disease promotes tau pathology in Alzheimer's disease. Brain Commun. 2020;2(2):fcaa132.
