# Supplementary figures and images for: Neuroprotective effect of the RNS60 in a mouse model of transient focal cerebral ischemia
Source: PLoS One. 2024 Jan 2;19(1):e0295504. doi: 10.1371/journal.pone.0295504 (PMC10760892; doi:10.1371/journal.pone.0295504)

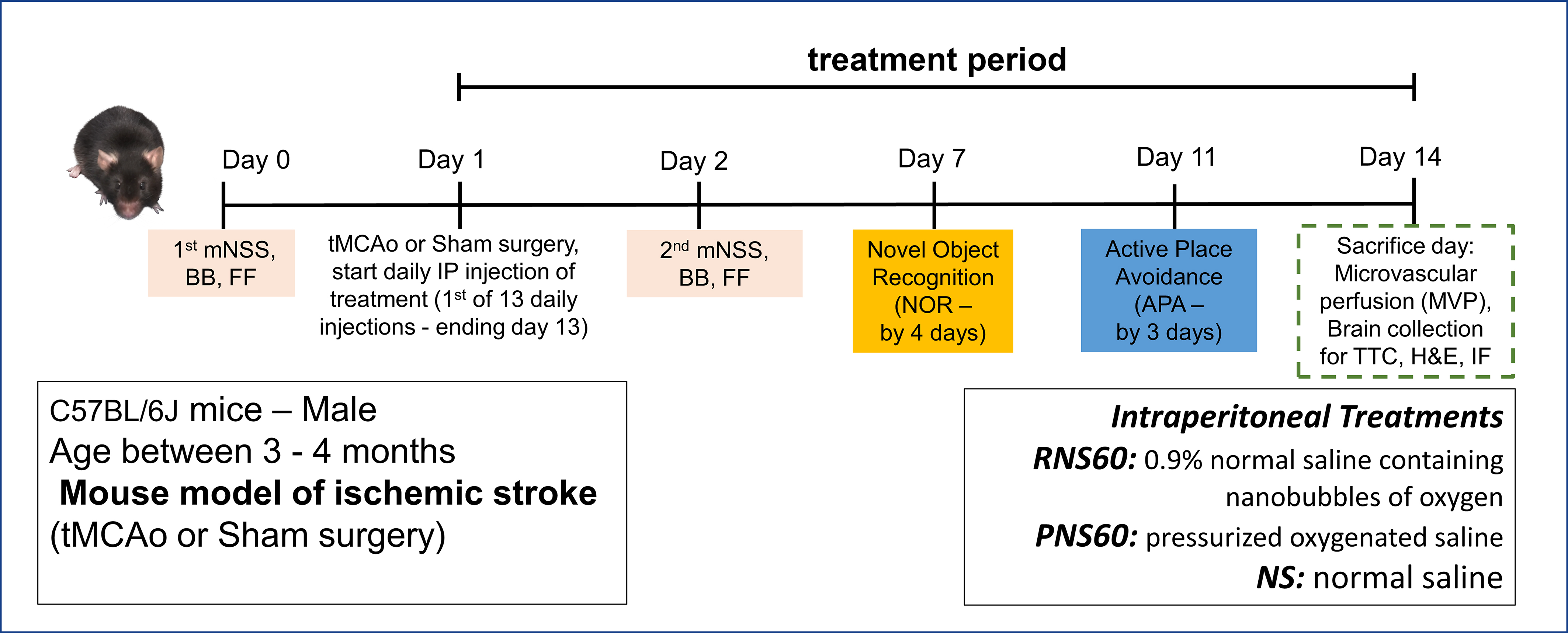

Supplement: S1 Fig — (TIF) [file pone.0295504.s003.tif]

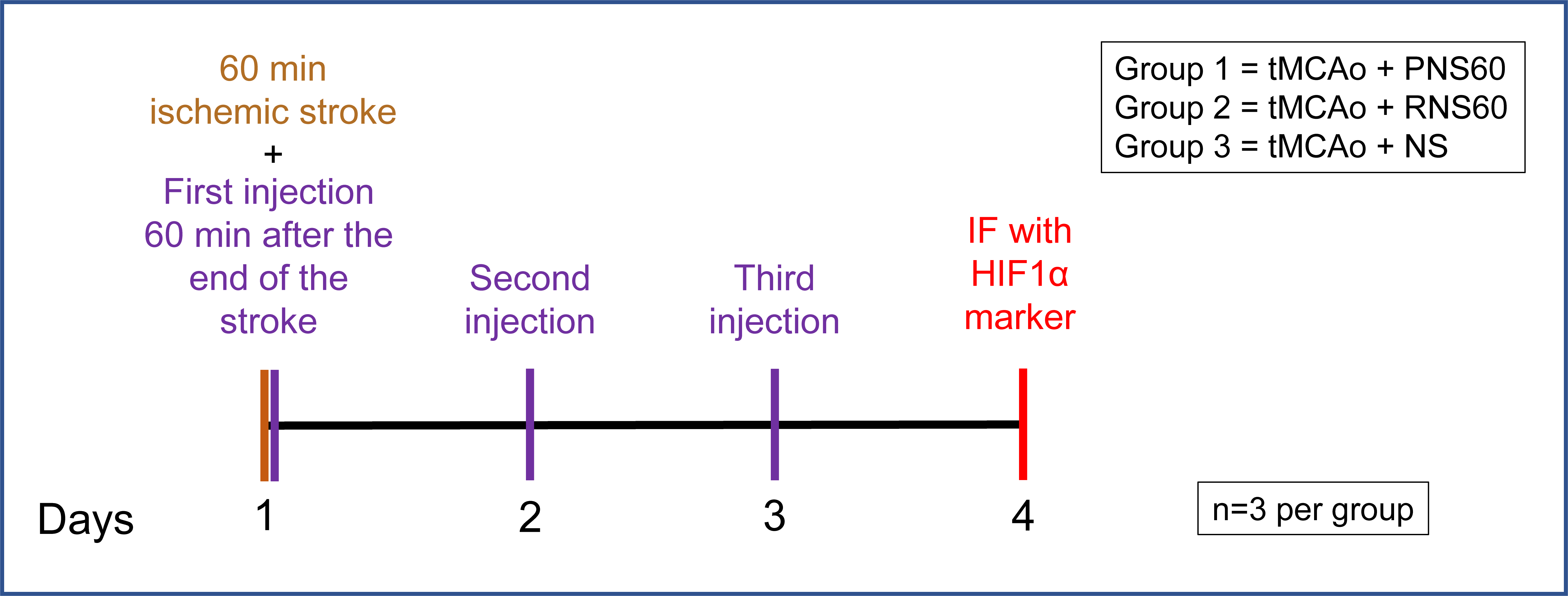

Supplement: S2 Fig — Animals with 1 h tMCAo treated with the different solutions and euthanized 4 days after stroke. The daily injections started one hour after tMCAo, ending the treatment on day 3 and euthanizing the animals on day 4. (TIF) [file pone.0295504.s004.tif]

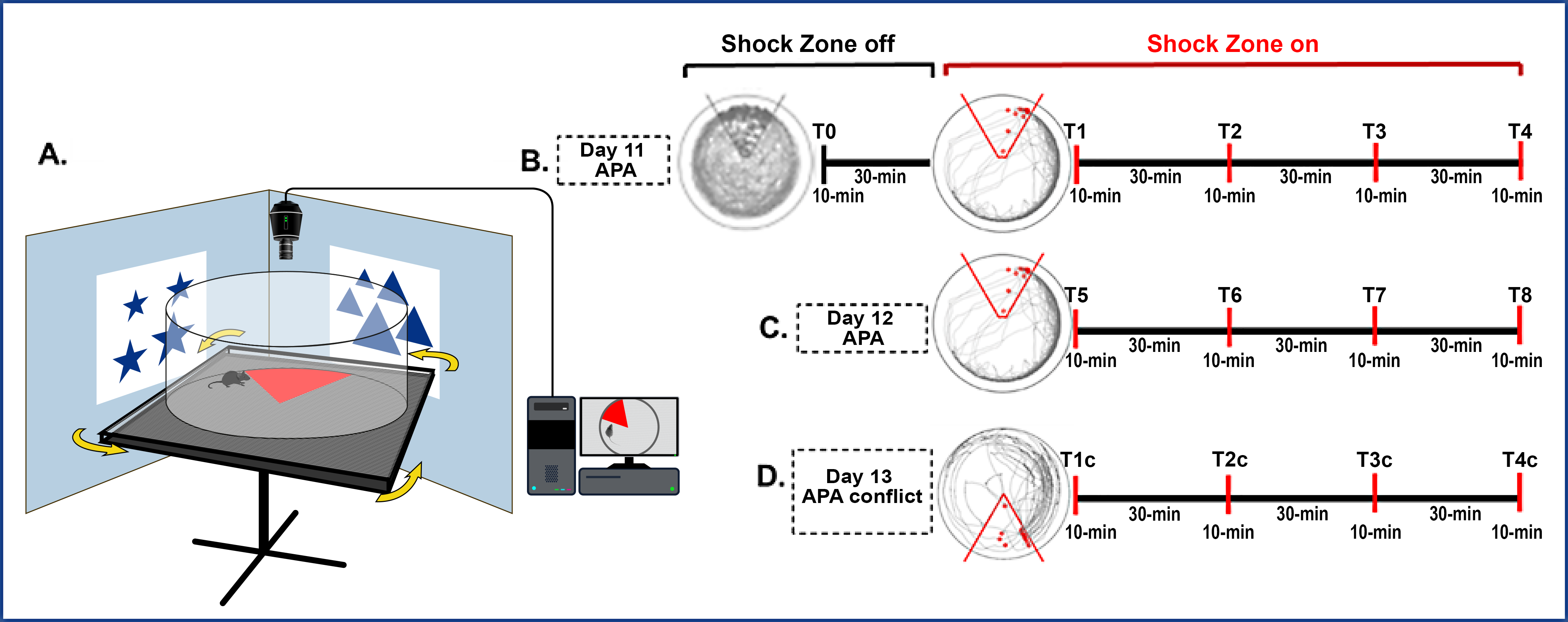

Supplement: S3 Fig — Tests were performed on days 11 (trials 1–4), 12 (trials 5–8) and 13 (APA conflict with trials 1–4) after tMCAo surgery. Training on days 11 and 12 assess regular learning (3B) and memory process (3C), whereas the day 13 test evaluates the mental flexibility (APA conflict), a form of cognitive discrimination evaluated by changing the location of the shock zone (3D). (TIF) [file pone.0295504.s005.tif]

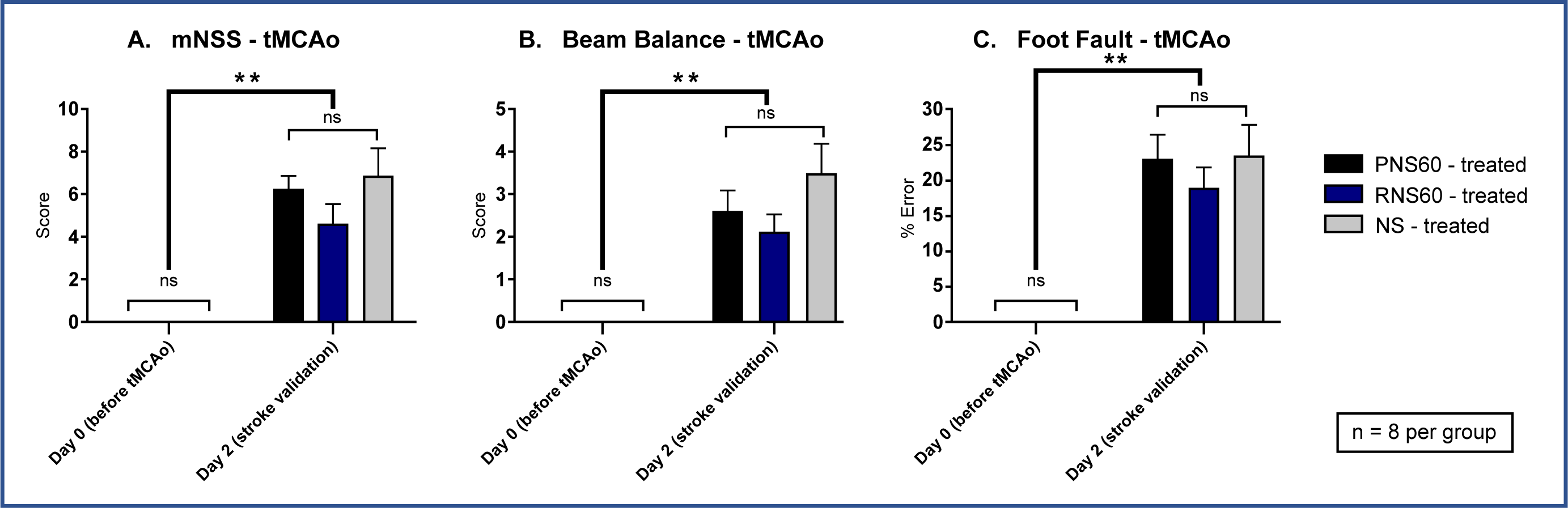

Supplement: S5 Fig — The values of the motor performance scores in the mNSS (A), BB (B) and FF (C) were expressed as mean ± SEM. Motor performance is affected 24-h after tMCAo (day 2) compared to 24 h before tMCAo (day 0). The graphs show a trend towards better performance by the RNS60 treated mice (blue bars) compared to mice treated with control solutions, without statistical significance. **p<0.01, ns = non-significant. (TIF) [file pone.0295504.s007.tif]

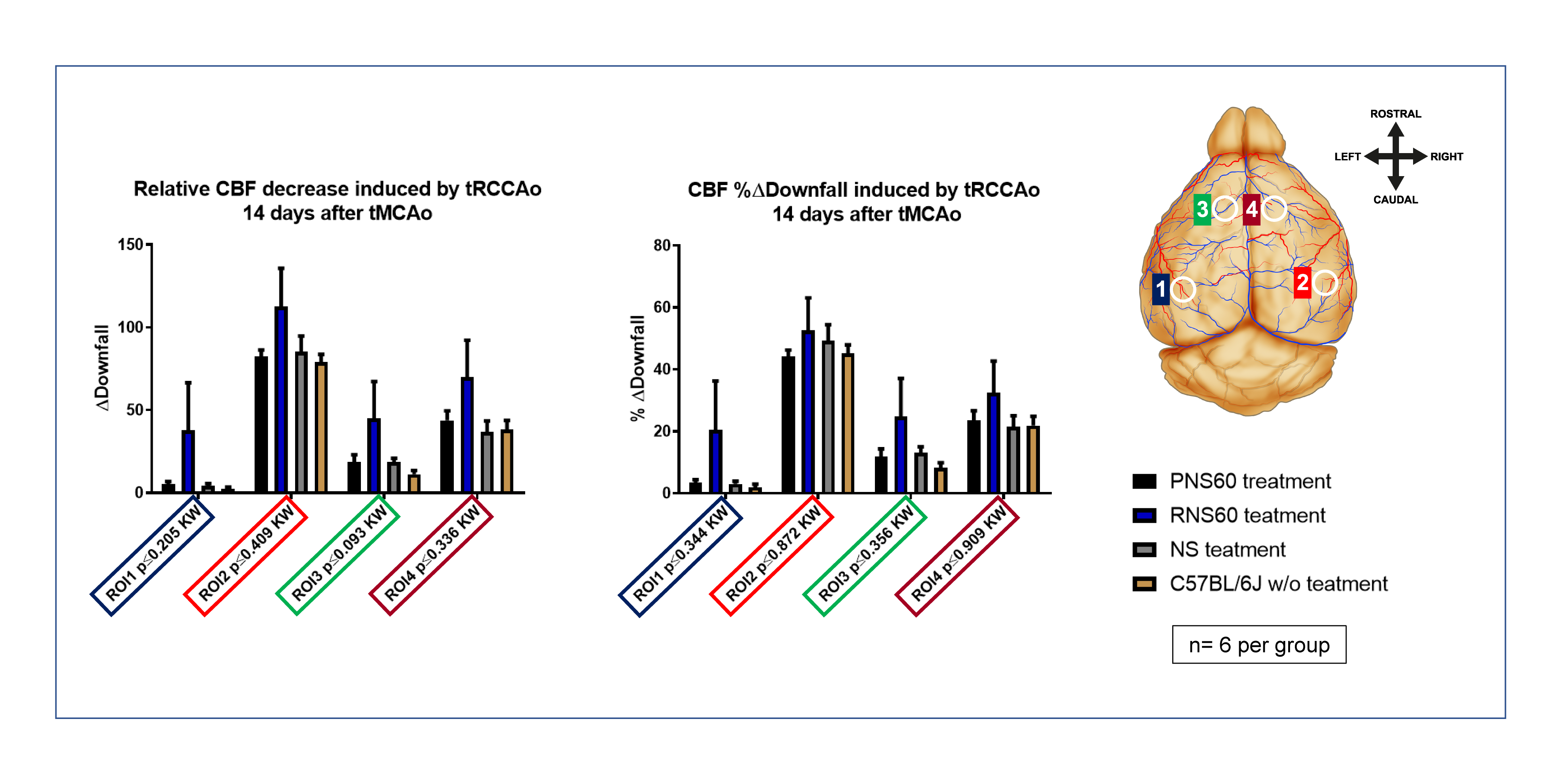

Supplement: S8 Fig — Measurements of CBF at day 14, show an increased trend of Δ Downfall and % Δ Downfall in animals treated with RNS60. This trend is more pronounced in ROI 1 and 3 of the Δ Downfall. (TIF) [file pone.0295504.s010.tif]

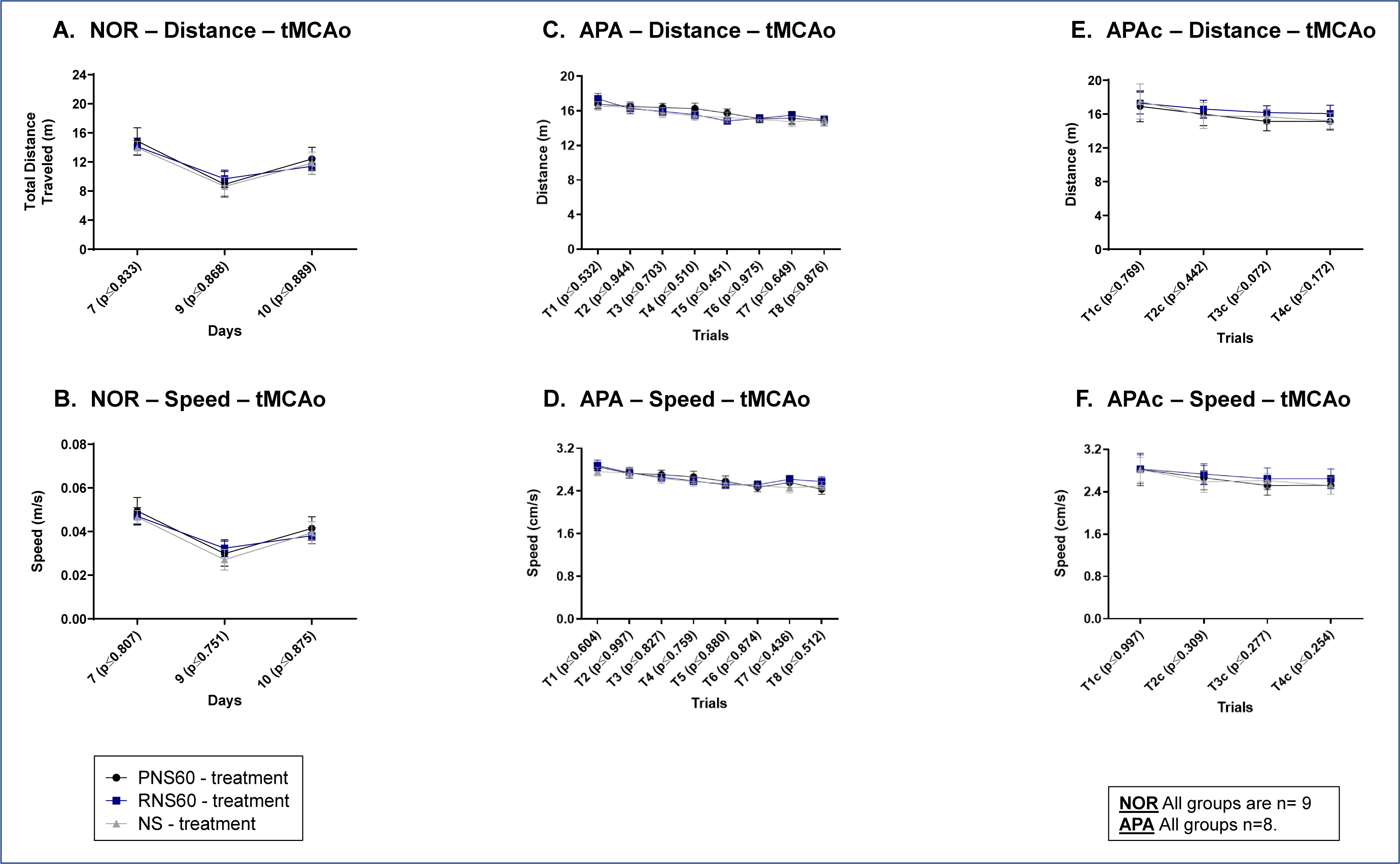

Supplement: S10 Fig — Animals going after tMCAo and treated with the different solutions (RNS60, PNS60 and NS) have no statistically significant differences in distance (A, C, E) and speed (B, D, F) when performing NOR (A, B) or APA (C-F), suggesting that the differences observed in Fig 2 are not due to motor skills differences. (TIF) [file pone.0295504.s012.tif]
